# Effectiveness of emerging technology-assisted rehabilitation in children with cerebral palsy: a systematic review and meta-analysis

**DOI:** 10.3389/fneur.2026.1906886

**Published:** 2026-09-09

**Authors:** Xintong Wei, Weichen Nie, Yuan Su, Chao Xie, Rui Chen, Yuanjun Zou, Tianying Chang

**Affiliations:** 1 College of Medical and Pharmaceutical Information, Changchun University of Chinese Medicine, Changchun, China; 2 College of Integrated Traditional Chinese and Western Medicine, Changchun University of Chinese Medicine, Changchun, China; 3 College of Basic Medical Sciences, Changchun University of Chinese Medicine, Changchun, China; 4 EBM Office, the Affiliated Hospital of Changchun University of Chinese Medicine, Changchun, China

**Keywords:** balance, cerebral palsy, emerging technology-assisted rehabilitation, gait, meta-analysis, robot-assisted gait training, virtual reality

## Abstract

**Introduction:**

Robot-assisted gait training (RAGT), virtual reality (VR), and other emerging technology-assisted interventions are increasingly used in pediatric neurorehabilitation, but their effects across functional domains and follow-up periods in children with cerebral palsy (CP) remain unclear.

**Methods:**

PubMed, Embase, the Cochrane Library, and Web of Science were searched from inception to February 23, 2026. Randomized controlled trials (RCTs) comparing these interventions with conventional rehabilitation in children with CP were included. Two reviewers independently screened studies, extracted data, and assessed risk of bias using the revised Cochrane Risk of Bias tool (RoB 2). Random-effects meta-analyses generated mean differences (MDs) with 95% confidence intervals (CIs). Subgroup analyses examined technology type and follow-up duration. The certainty of evidence for each outcome was assessed using the Grading of Recommendations Assessment, Development and Evaluation (GRADE) approach.

**Results:**

Forty-six RCTs involving 1,812 children were included. Emerging technology-assisted interventions improved performance on the 10-Meter Walk Test (10MWT; MD = −2.83, 95% CI −4.39 to −1.27), 6-Minute Walk Test (6MWT; MD = 43.56, 95% CI 25.55 to 61.57), Timed Up and Go Test (TUG; MD = −2.17, 95% CI −3.60 to −0.75), Pediatric Balance Scale (PBS; MD = 5.21, 95% CI 4.06 to 6.37), Gross Motor Function Measure (GMFM) dimension D (MD = 3.99, 95% CI 1.61 to 6.37), and GMFM dimension E (MD = 8.90, 95% CI 5.18 to 12.62). RAGT favored gait outcomes, whereas VR favored balance outcomes. Upper-limb spasticity measured by the Modified Ashworth Scale (MAS) decreased (MD = −0.40, 95% CI −0.59 to −0.21). ABILHAND-Kids questionnaire scores showed no immediate improvement (MD = 2.00, 95% CI −1.01 to 5.01) but improved at ≥6 weeks (MD = 2.54, 95% CI 0.41 to 4.66). Most gains were immediate; only balance and manual ability remained significant at longer follow-up.

**Conclusion:**

Emerging technology-assisted rehabilitation improves gait, mobility, balance, gross motor function, and upper-limb outcomes in children with CP. RAGT and VR show domain-specific advantages. Evidence certainty ranged from high to very low, and further high-quality RCTs with extended follow-up are needed.

**Systematic review registration:**

https://www.crd.york.ac.uk/PROSPERO/view/CRD420251157757, Identifier CRD420251157757.

## 1 Introduction

Cerebral palsy (CP) describes a group of permanent disorders of the development of movement and posture attributed to non-progressive disturbances occurring in the fetal or infant brain, with activity limitation constituting its cardinal feature (1–3). As a leading cause of childhood physical disability worldwide (4), CP frequently co-occurs with sensory, cognitive, communication, and other impairments (5–9), which collectively impede motor development and daily activities, thereby compromising quality of life and long-term prognosis (10–13).

The primary goals of rehabilitation for CP are to improve motor function and enhance independence in daily living. Task-specific training (TST) and task-oriented training (TOT) represent core evidence-based interventions for this population (14–16). TST emphasizes active, repetitive practice of a clearly defined motor task, such as reaching or walking, whereas TOT focuses on meaningful, goal-directed functional activities, such as dressing or transfers, in contexts relevant to daily life (15, 17). Other commonly used rehabilitation approaches include neurodevelopmental treatment (NDT), treadmill training (TT), passive stretching, and other physical therapy modalities (18–20). NDT is an individualized, therapist-guided approach that uses movement analysis, therapeutic handling, and facilitation to influence postural control, selective movement, and functional task performance (21). However, meta-analytic evidence indicates that NDT does not outperform activity-based alternatives in improving motor function among children with CP (22). TT involves repetitive stepping or walking practice on a treadmill (23), while passive stretching involves externally applied force or sustained positioning to maintain or increase joint range of motion (24). Although TT may improve walking speed and passive stretching may provide short-term gains in joint range of motion, evidence for sustained functional benefits from either approach remains limited (25, 26). Overall, conventional rehabilitation may be constrained by insufficient training intensity, suboptimal patient engagement, limited objective feedback, and dependence on therapist expertise (27).

The convergence of digital health technologies and neurorehabilitation has given rise to a spectrum of emerging interventions for CP rehabilitation (28, 29). Robot-assisted gait training (RAGT) provides high-intensity, repetitive practice that may promote sensorimotor remodeling and neuroplasticity (30–32). Virtual reality (VR) and augmented reality (AR) technologies provide interactive training and real-time multimodal feedback, which may enhance engagement and treatment adherence (33–35). Metaverse-based rehabilitation platforms use immersive virtual scenarios and posture recognition to support training and quantitative assessment (36). Collectively, these technologies may support sensorimotor learning, neuroplastic adaptation, and individualized rehabilitation.

Current evidence from systematic reviews and meta-analyses remains mixed. Several reviews have reported potential benefits of VR-assisted training and RAGT for gross motor function, balance, lower-limb function, and functional independence (37–40), whereas other syntheses have found the evidence for RAGT and exoskeleton-assisted rehabilitation to be inconsistent or inconclusive (41–44). These syntheses are further constrained by small sample sizes in the included trials, substantial heterogeneity, and limited certainty of evidence (45–47), while evaluations of treatment durability across follow-up intervals remain scarce (32, 48). Most reviews have also focused on individual technologies, limiting integrated comparisons across functional domains and intervention types.

To address these gaps, this systematic review and meta-analysis synthesized evidence from randomized controlled trials (RCTs) comparing emerging rehabilitation technologies with conventional rehabilitation in children with CP. We evaluated upper- and lower-extremity motor function, gait, balance, and functional independence, and assessed treatment durability across short-term (< 6 weeks) and medium- to long-term (≥ 6 weeks) follow-up intervals. The aim was to inform individualized rehabilitation strategies for children with CP.

## 2 Materials and methods

### 2.1 Study design

This systematic review and meta-analysis was conducted according to the methodological guidance of the Cochrane Handbook for Systematic Reviews of Interventions (49) and reported in accordance with the Preferred Reporting Items for Systematic Reviews and Meta-Analyses (PRISMA) 2020 statement (50). The review protocol was prospectively registered in the International Prospective Register of Systematic Reviews (PROSPERO CRD420251157757).

### 2.2 Data sources and search strategy

Two reviewers (YS, CX) independently conducted systematic literature searches across four electronic databases: PubMed, Web of Science (WOS), the Cochrane Library, and Embase. The searches encompassed records from the inception of each database through February 23, 2026. To minimize the risk of omission, the search scope was supplemented by hand-searching reference lists of eligible articles, screening gray literature, and tracking bibliographies of relevant systematic reviews. Discrepancies were resolved through consultation with a third reviewer (TC).

The search strategy was developed in accordance with the PICOS framework, as recommended by the Cochrane Handbook (49), with detailed eligibility criteria presented in Section 2.3. In brief, the strategy employed a combination of controlled vocabulary and free-text terms, tailored to the thesaurus structure of each database (e.g., MeSH for PubMed, Emtree for Embase). Boolean operators (AND/OR) and database-specific search tags were applied to optimize keyword matching. Core search terms included, but were not restricted to, “cerebral palsy,” “CP,” “spastic cerebral palsy,” “dyskinetic cerebral palsy,” “mixed cerebral palsy,” “robotics,” “virtual reality,” “augmented reality,” “brain-computer interface,” “digital health,” and “intelligent systems.” Detailed search strategies are provided in Appendix S1. No language, date, or full-text restrictions were applied.

### 2.3 Inclusion and exclusion criteria

#### 2.3.1 Inclusion criteria

Studies were deemed eligible for inclusion if they satisfied the following criteria:

1. Population: Children with a clinical diagnosis of CP, irrespective of race or sex, aged ≤18 years.
2. Intervention: Emerging rehabilitation technologies, including but not limited to RAGT, VR, AR, digital health applications, and wearable sensor systems.
3. Comparison: Control groups receiving conventional intervention protocols, such as conventional physical therapy, treadmill training, or standard traditional rehabilitation.
4. Outcomes: Studies were required to report at least one outcome measure of interest, spanning lower-extremity motor function and gait, upper-extremity motor function and fine motor activity, and global motor function and participation. Detailed outcome measures are specified in Section 2.4.
5. Study Design: RCTs. Studies were eligible when explicitly described as RCTs with prospective random allocation to intervention and control groups validated in the full-text methods; trial registration records were also reviewed for confirmation if available. The RCT status of each study was independently verified by two reviewers (XW, WN) during literature screening.

#### 2.3.2 Exclusion criteria

Studies were excluded from the review if they met any of the following conditions: (1) were observational studies, case–control studies, crossover RCTs, narrative reviews, systematic reviews or meta-analyses, editorials, commentaries, conference abstracts, or other non-empirical publications; (2) were duplicate publications or of inadequate methodological quality; (3) had irretrievable full texts or lacked extractable outcome data; (4) involved experimental interventions outside the predefined scope of emerging rehabilitation technologies, or co-administered adjunctive treatments such as botulinum toxin injections or orthopedic surgery, where the isolated effect of the emerging technology could not be extracted.

### 2.4 Outcome measures

The outcome measures of interest in this review were categorized into the following three functional domains:

(1) Lower-extremity motor function and gait: 10-Meter Walk Test (10MWT), 6-Minute Walk Test (6MWT), Timed Up and Go (TUG) test, Gross Motor Function Measure (GMFM) total score and Dimension D (Standing) and Dimension E (Walking, Running, and Jumping) subscores, Pediatric Balance Scale (PBS), Modified Ashworth Scale lower-extremity score (MAS–Lower Limb), and Functional Independence Measure for Children (WeeFIM) Lower Extremity total score and Self-Care subscale scores.
(2) Upper-extremity motor function and fine motor activity: Modified Ashworth Scale upper-extremity score (MAS-Upper Limb), Quality of Upper Extremity Skills Test (QUEST) total score and Dissociated Movement and Grasp subscores, ABILHAND-Kids questionnaire, Box and Block Test (BBT), and Assisting Hand Assessment (AHA).
(3) Global motor function and participation: WeeFIM total score and Canadian Occupational Performance Measure (COPM) performance score.

### 2.5 Study Selection and Data Extraction

All references were imported into EndNote X9, and two independent reviewers (XW, WN) assessed eligibility in a blinded manner. Study selection proceeded in two stages: titles and abstracts were screened first, after which full-text articles were retrieved and evaluated against the eligibility criteria; discrepancies were resolved through adjudication by a third reviewer (YZ). Data from each eligible study were extracted using a standardized, piloted form. Extracted information encompassed study characteristics including first author, year of publication, country, sample size, CP subtype, and participant age; intervention characteristics including technology category, device model, protocol, session duration, frequency, and total intervention period; and outcome data including means and standard deviations for all measures specified in Section 2.4, at both immediate post-intervention and follow-up time points. Missing standard deviations were estimated from standard errors, ranges, interquartile ranges, or medians using established transformation methods (49, 50).

### 2.6 Risk of Bias assessment

Two reviewers (XW, WN) independently assessed the risk of bias of all included RCTs using the revised Cochrane Risk of Bias tool (RoB 2.0) (51). A third reviewer (RC) verified domain classifications and resolved discrepancies. The assessment encompassed five domains: bias from randomization, deviations from intended interventions, missing outcome data, measurement of the outcome, and selection of the reported result. Each domain was rated as “low risk of bias,” “some concerns,” or “high risk of bias.”

### 2.7 Data synthesis and statistical analysis

All meta-analyses were performed by two reviewers (XW, WN) using RevMan 5.4. Quantitative synthesis was conducted when comparative data were available from at least two independent studies. A random-effects model was used to account for anticipated clinical and methodological heterogeneity in intervention protocols, participant characteristics, and outcome assessment methods. Data were pooled using the DerSimonian and Laird (52) method. Effect sizes were expressed as mean differences (MDs) with 95% confidence intervals (CIs), and forest plots were generated for all analyses (53). Each functional outcome was quantified using identical standardized rehabilitation scales across trials. We therefore calculated MD to report absolute clinical changes, as MD preserves the native scale units for direct clinical interpretation. Between-study heterogeneity was assessed using the Q test (*p* < 0.1 indicating heterogeneity) and I^2^ statistic, classified as low (<25%), moderate (25% ≤ I^2^ ≤ 50%), or substantial (>50%) (54). Publication bias was evaluated using Stata 18.0 for outcomes with ≥10 studies, using Egger’s test (*p* < 0.1 indicating potential bias) (55), funnel plot inspection, and the trim-and-fill method (56).

Prespecified subgroup analyses stratified studies by technology type and follow-up duration (short-term < 6 weeks and medium- to long-term ≥ 6 weeks), for lower-extremity, upper-extremity, and global motor outcomes. Sensitivity analyses were conducted using a leave-one-out approach for outcomes with substantial heterogeneity (I^2^ > 50%).

### 2.8 Assessment of the certainty of the evidence

Two reviewers (XW, WN) independently assessed the certainty of evidence for each outcome using the Grading of Recommendations Assessment, Development and Evaluation (GRADE) approach (57). Evidence from RCTs was initially rated as high certainty and was downgraded by one or two levels, as appropriate, for concerns regarding risk of bias, inconsistency, indirectness, imprecision, or publication bias. Disagreements between the reviewers were resolved through discussion and consensus. The final certainty of evidence was classified as high, moderate, low, or very low.

## 3 Results

### 3.1 Search results

**Figure 1:**
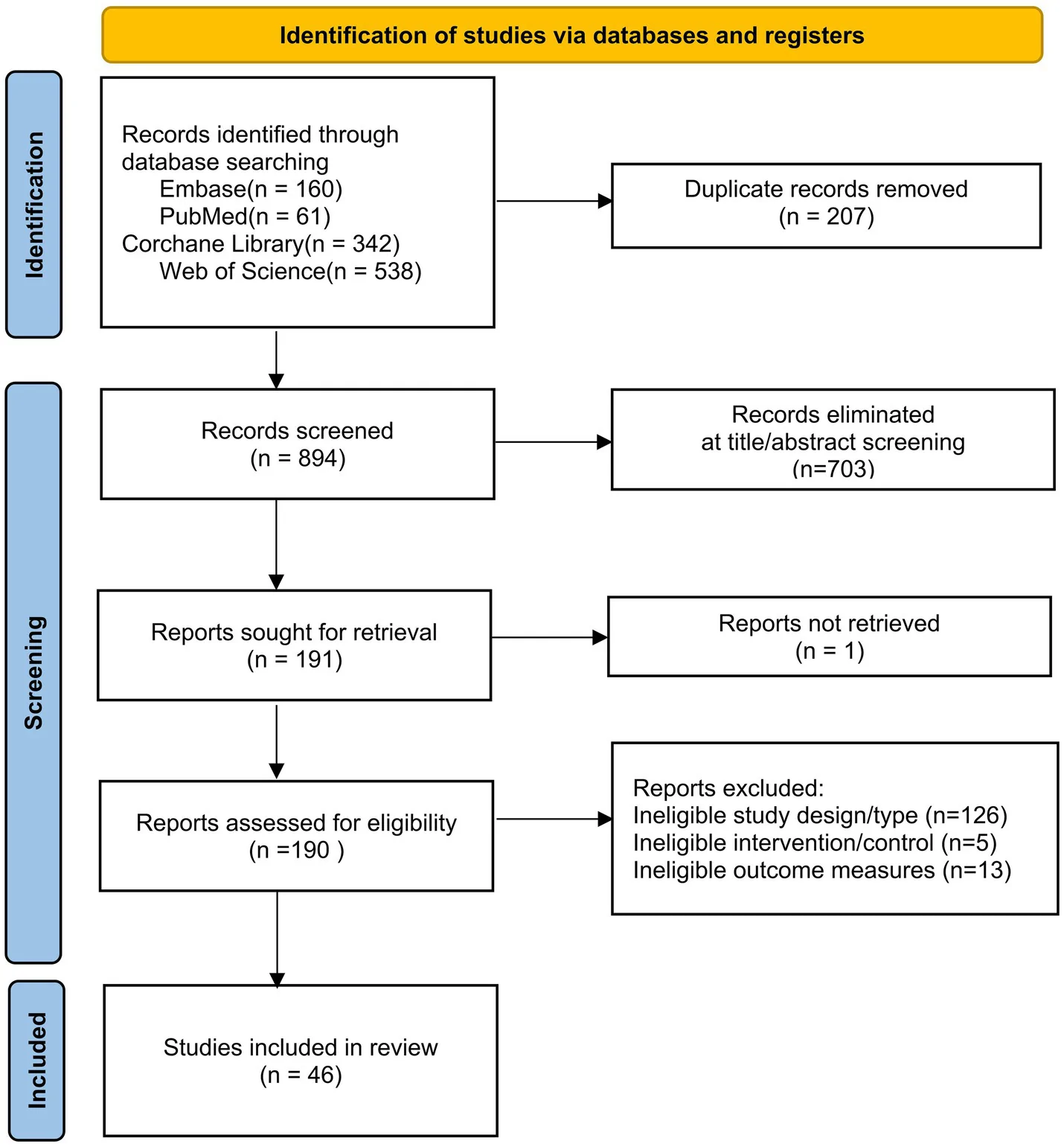
PRISMA 2020 flow diagram for study selection.

The initial systematic search yielded 1,101 records, comprising 61 from PubMed, 538 from Web of Science, 342 from the Cochrane Library, and 160 from Embase. After 207 duplicate records were removed, 894 records underwent title and abstract screening, of which 703 were eliminated at this stage. Of the remaining 191 reports sought for retrieval, the full texts of 190 reports were successfully obtained, while one report could not be retrieved despite reasonable efforts. The 190 retrieved reports were subsequently assessed for eligibility through full-text review, and 144 were excluded because of an ineligible study design (*n* = 126), an ineligible intervention or control (*n* = 5), or ineligible outcome measures (*n* = 13). Ultimately, 46 studies met the inclusion criteria and were included in the systematic review and meta-analysis (58–103). The study selection process is summarized in the PRISMA flow diagram presented in Figure 1.

### 3.2 Characteristics of included studies

A total of 46 independent RCTs were included in this systematic review. Publication dates ranged from 2011 to 2025. The included studies were conducted across multiple countries: Australia (82, 96), Belgium (74, 90, 98), Brazil (86), China (including Taiwan) (80, 81, 95, 100, 102), Egypt (66, 67, 73, 91), France (64, 69), Germany (79), India (61, 62, 84, 93), Iran (63), Italy (103), South Korea (71, 75, 77, 83, 85), Lebanon (65), Poland (101), Saudi Arabia (88, 99), Turkey (58, 59, 68, 72, 76, 78, 87, 89, 94), and the United States (60, 92).

The 46 trials enrolled a total of 1,812 children with CP. Among the 29 studies that reported sex distribution, 600 participants were male and 540 were female; the remaining studies did not specify sex breakdown. The mean age of participants was 9.24 ± 3.17 years. All participants had a confirmed diagnosis of CP, with spastic subtypes predominating—primarily spastic hemiplegia and spastic diplegia, with a smaller subset of studies including tetraplegia and other forms. According to the Gross Motor Function Classification System (GMFCS), the vast majority of participants were classified at levels I to III, with only a limited number of studies enrolling children at levels IV or V. A total of 932 participants were allocated to experimental groups receiving emerging technologies, and 880 participants were assigned to control groups receiving conventional therapy. Detailed characteristics of all included studies are presented in Table 1.

**Table 1:** Characteristics of the studies included in the systematic review and meta-analysis.

Study	Funding	*N*	M/F	CP Type	TC	FC (GMFCS/MACS)	Groups	Age (Years)	Evaluation	Outcome measures
Tekeş et al. (59) (Turkey)	No	57	28/29	spastic	Hemiplegia = 20 Bilateral = 37	GMFCS II-IV	EG = 28 CG = 29	9.55 ± 3.59	T1 Baseline T2 Post-intervention T3 Follow-up (6 weeks) T4 Follow-up (12 weeks)	GMFM-D, E, Total 6MWT 10MWT PedsQoL
Julien et al. (69) (France)	Yes	40	19/21	spastic	Hemiplegia = 40	GMFCS-ER I-II	EG = 20 CG = 20	8.4 ± 3.5	T1 Baseline T2 Follow-up (4 weeks)	GMFM-D, E 6MWT
Hui et al. (70) (China)	No	40	NR	spastic	Hemiplegia = 20 Bilateral = 20	GMFCS I-III	EG = 20 CG = 20	5.6 ± 1.9	T1 Baseline T2 Post-intervention	GMFM-D, E 6MWT 10MWT PBS; MAS
Choi et al. (71) (South Korea)	Yes	90	49/41	NR	Hemiplegia Bilateral	GMFCS II-IV	EG = 45 CG = 45	9.51 ± 2.48	T1 Baseline T2 Post-intervention T3 Follow-up (4 weeks)	GMFM-D, E, Total; PBS 6MWT Cadence; Walking speed; stride length; Step width
Adar et al. (72) (Turkey)	No	19	10/9	NR	Hemiplegia = 7 Bilateral = 12	GMFCS II-III	EG = 9 CG = 10	9.74 ± 2.25	T1 Baseline T2 Post-intervention	FMA-UE, Hand BBT ABILHAND-Kids WeeFIM
Yaśar et al. (78) (Turkey)	No	26	13/13	spastic	Bilateral = 26	GMFCS II-V	EG = 13 CG = 13	10.08 ± 2.55	T1 Baseline T2 Post-intervention	TUG WeeFIM
Moll et al. (79) (Germany)	Yes	30	23/7	spastic	Tetraparesis = 27 Diparesis = 7	GMFCS II-III	EG = 15 CG = 15	13.0 ± 2.5	T1 Baseline T2 Post-intervention	GMFM-Total 6MWT
Pool et al. (82) (Australia)	Yes	40	22/18	NR	NR	GMFCS III-V	EG = 20 CG = 20	8.08 ± 2.08	T1 Baseline T2 Post-intervention T3 Follow-up (20 weeks)	WeeFIM Totle, Self-care, Mobility; COPM Performance, Satisfaction GMFM-Total;
Wallard et al. (90) (Belgium & France)	No	30	15/15	Spastic	Diplegic	GMFCS II	EG = 14 CG = 16	9.00 ± 1.49	T1 Baseline T2 Post-intervention	GMFM-D, E Cadence Walking speed
El-Shamy, (91) (Egypt)	No	30	20/10	spastic	Hemiplegia = 30	MACS I-III	EG = 15 CG = 15	6.85 ± 0.79	T1 Baseline T2 Post-intervention	MAS QUEST Total score, Dissociated movement, Grasp., Weight bearing, Protective extension
Wu et al. (92) (United States)	Yes	23	14/9	Spastic	Diplegia = 8 Quadriplegia = 14 Triplegia = 1	GMFCS I-IV	EG = 11 CG = 12	10.9 ± 3.2	T1 Baseline T2 Post-intervention T3 Follow-up (8 weeks)	6MWT GMFM- D, E, Total MAS
Gilliaux et al. (98) (Belgium)	Yes	16	7/9	NR	Hemiplegia = 1 Diplegia = 8 Quadriplegia = 7	MACS II-IV	EG = 8 CG = 8	10.9 ± 4.09	T1 Baseline T2 Post-intervention	BBT QUEST-Dissociated movement, Grasp Abilhand-Kids
Sarhan et al. (99) (Saudi Arabia)	Yes	12	7/5	Spastic	Bilateral	GMFCS III-IV	EG = 6 CG = 6	4.2 ± 0.7	T1 Baseline T2 Post-intervention	stride length cadence gait velocity
Druzbicki et al. (101) (Poland)	No	52	NR	Spastic	Bilateral	GMFCS II-III	EG = 26 CG = 26	10.33 ± 2.23	T1 Baseline T2 Post-intervention	gait velocity Step width
Smania et al. (103) (Italy)	Yes	18	10/8	Spastic	Diplegia Tetraplegia	GMFCS II-IV	EG = 9 CG = 9	13.34 ± 2.96	T1 Baseline T2 Post-intervention T3 Follow-up (4 weeks)	6MWT 10MWT WeeFIM-total score
Peramalaiah et al. (61) (India)	Yes	34	18/16	NR	Hemiplegia Bilateral	GMFCS I-III	EG = 17 CG = 17	7.80 ± 2.65	T1 Baseline T2 Post-intervention	PDMS-2
Karabulut et al. (68) (Turkey)	No	30	NR	Spastic	Hemiplegia	GMFCS I-III	EG = 15 CG = 15	8.75 ± 3.95	T1 Baseline T2 Post-intervention	Abilhand-Kids
Abd-Elfattah et al. (73) (Egypt)	No	60	33/27	Spastic	Hemiplegic = 60	GMFCS I-II	EG = 30 CG = 30	6.04 ± 0.78	T1 Baseline T2 Post-intervention	QUEST-Dissociated movement, Grasp., Weight bearing, Protective reaction Nine-Hole Peg Test
Wang et al. (81) (Taiwan, China)	Yes	20	NR	Spastic	Hemiplegic = 20	MACS I-III	EG = 10 CG = 10	8.56 ± 2.09	T1 Baseline T2 Post-intervention	Abilhand-Kids WeeFIM -self care
Jung et al. (83) (South Korea)	No	10	5/5	Spastic	Diplegic = 10	GMFCS I-II	EG = 5 CG = 5	12.40 ± 2.12	T1 Baseline T2 Post-intervention	PBS Velocity Cadence
El-Shamy et al. (88) (Saudi Arabia)	No	40	26/14	Spastic	Hemiplegic = 40	MACS I-III	EG = 20 CG = 20	9.65 ± 1.30	T1 Baseline T2 Post-intervention	PDMS-2 MAS
Sajan et al. (93) (India)	No	20	11/9	Spastic	Diplegic = 1 Triplegic = 3 Quadriplegic = 13	GMFCS I-IV	EG = 10 CG = 10	11.50 ± 4.39	T1 Baseline T2 Post-intervention	PBS, BBT QUEST Grasp., Total score Walking speed
Tarakci et al. (94) (Turkey)	No	38	NR	Spastic Dyskinetic	Hemiplegic = 14 Diplegic = 12	GMFCS I-III	EG = 19 CG = 19	10.50 ± 2.74	T1 Baseline T2 Post-intervention	10MWT WeeFIM Totle, Self-care, Transfers, Locomotion TUG
Chiu et al. (100) (Taiwan, China)	Yes	62	28/34	Spastic	Hemiplegic = 62	GMFCS I-V	EG = 32 CG = 30	9.45 ± 1.90	T1 Baseline T2 Post-intervention T3 Follow-up (6 weeks)	Grip Strength Nine-hole Peg Test
Yenilmez et al. (58) (Turkey)	No	34	NR	Spastic	Hemiplegic = 34	GMFCS I-II	EG = 17 CG = 17	10.67 ± 3.21	T1 Baseline T2 Post-intervention	GMFM-D, E TUG
Roberts et al. (60) (United States)	Yes	33	13/20	Spastic	Hemiplegic = 33	GMFCS I-II	EG = 13 CG = 20	9.25 ± 3.08	T1 Baseline T2 Post-intervention	AHA COPM Performance
Dwarkadas et al. (62) (India)	Yes	32	NR	Spastic	Diplegic = 32	GMFCS II	EG = 16 CG = 16	10.4 ± 2.08	T1 Baseline T2 Post-intervention	PBS
Daliri et al. (63) (Iran)	Yes	30	15/15	Spastic	Hemiplegic = 30	GMFCS I-III	EG = 15 CG = 15	7.40 ± 1.19	T1 Baseline T2 Post-intervention T3 Follow-up (6 weeks)	Grip Strength
Baillet et al. (64) (France)	Yes	22	NR	Spastic Dyskinetic	Hemiplegic = 13 Quadriplegic = 7	GMFCS II-IV	EG = 10 CG = 12	14.5 ± 2.1	T1 Baseline T2 Post-intervention T3 Follow-up (12 weeks)	BBT
Ziab et al. (65) (Lebanon & Iran)	No	46	NR	Spastic	Hemiplegic = 23 Diplegic = 17 Monoplegic = 3	GMFCS I-II	EG1 = 14 EG2 = 14 CG = 18	7.86 ± 2.67	T1 Baseline T2 Post-intervention T3 Follow-up (6 weeks)	GMFM-D, E PBS
Mouhamed et al. (66) (Egypt & Saudi Arabia)	No	64	NR	Ataxic	NR	GMFCS I-II	EG = 32 CG = 32	10.94 ± 1.32	T1 Baseline T2 Post-intervention	PBS
Madboly et al. (67) (Egypt)	No	75	31/44	Spastic	Hemiplegic = 75	GMFCS I-II	EG1 = 25 EG2 = 25 CG = 25	9.15 ± 1.18	T1 Baseline T2 Post-intervention	6MWT
Saussez et al. (74) (Belgium & France)	Yes	40	NR	Spastic	Hemiplegic = 40	GMFCS I-II	EG = 20 CG = 20	9.05 ± 3.00	T1 Baseline T2 Post-intervention T3 Follow-up (12 weeks)	AHA; BBT 6MWT ABILHAND-Kids PEDI; COPM Performance, Satisfaction
Menekseoglu et al. (76) (Turkey)	Yes	38	20/18	Spastic	Hemiplegic = 38	GMFCS I-II	EG = 19 CG = 19	8.3 ± 1.6	T1 Baseline T2 Post-intervention T3 Follow-up (12 weeks)	AHA ABILHAND-Kids QUEST total score
Choi et al. (77) (South Korea)	Yes	40	NR	NR	NR	NR	EG = 20 CG = 20	7.6 ± 2.9	T1 Baseline T2 Post-intervention T3 Follow-up (6 weeks)	MA2-Range, Accuracy PEDI-CAT-Social Cognitive
Jha et al. (84) (India)	No	38	23/15	Spastic	Diplegic = 38	GMFCS II-III	EG = 19 CG = 19	8.83 ± 1.8	T1 Baseline T2 Post-intervention T3 Follow-up (8 weeks)	PBS GMFM-Total WeeFIM
Choi et al. (85) (South Korea & China)	Yes	80	NR	NR	NR	MACS I-IV	EG = 40 CG = 40	5.7 ± 2.8	T1 Baseline T2 Post-intervention T3 Follow-up (8 weeks)	MA-2-Range, Accuracy, Fluency PEDI-CAT-Daily Activity, Mobility, Social Cognitive, Responsibility ROM
Arnoni et al. (86) (Brazil)	Yes	22	11/11	Spastic	Hemiplegic = 22	GMFCS I	EG = 11 CG = 11	8.4 ± 2.3	T1 Baseline T2 Post-intervention	TUG Velocity
Sahin et al. (87) (Turkey)	No	60	37/23	Spastic	Hemiplegic = 60	GMFCS I-III	EG = 30 CG = 30	10.3 ± 3.4	T1 Baseline T2 Post-intervention	WeeFIM Totle, Self-care, Locomotion, Transfer, Social Cognition, Sphincter Control
Okmen et al. (89) (Turkey)	No	46	NR	Spastic = 39 Dyskinetic = 1 Mixed = 1	Diplegic = 27 Quadriplegic = 9 Triplegic = 1 Hemiplegic = 4	GMFCS II-IV	EG = 23 CG = 23	8.5 ± 2.2	T1 Baseline T2 Post-intervention	BFMF GMFCS
Ren et al. (95) (China)	Yes	40	NR	Spastic	Diplegic = 40	GMFCS I-II	EG = 20 CG = 20	4.6 ± 1.0	T1 Baseline T2 Post-intervention	GMFM-D, E MAS; BBS PDMS-2-Grip, Visual-Motor Integration, Fine Motor Development Quotient
Cho et al. (97) (South Korea)	No	18	NR	Spastic	NR	GMFCS I-III	EG = 9 CG = 9	9.8 ± 3.6	T1 Baseline T2 Post-intervention	GMFM-D, E PBS 10MWT
Chen et al. (102) (Taiwan, China)	Yes	30	NR	Spastic	Diplegic = 19 Hemiplegic = 8	GMFCS I-II	EG = 14 CG = 16	8.7 ± 2.2	T1 Baseline T2 Post-intervention	GMFM-Total
Fu et al. (80) (China)	Yes	60	28/32	Spastic	NR	GMFCS II-III	EG1 = 15 EG2 = 15 EG3 = 15 CG = 15	5.1 ± 2.1	T1 Baseline T2 Post-intervention	GMFM-D, E 6MWT MAS
Mitchell et al. (96) (Australia)	Yes	101	52/49	NR	Hemiplegic = 101	GMFCS I-II	EG = 51 CG = 50	11.3 ± 2.4	T1 Baseline T2 Post-intervention	6MWT
Moon et al. (75) (South Korea)	No	26	12/14	Spastic	NR	GMFCS II-III	EG = 13 CG = 13	16.8 ± 3.0	T1 Baseline T2 Post-intervention	GMFM-Total PedsQoL

ABILHAND-Kids, ABILHAND-Kids Questionnaire; AHA, Assisting Hand Assessment; BBT, Box and Block Test; BBS, Berg Balance Scale; BFMF, Brief Functional Motor Function Scale; CG, Control Group; COPM, Canadian Occupational Performance Measure; CP, Cerebral Palsy; EG, Experimental Group; FC, Functional Classification; FMA-UE, Fugl-Meyer Assessment of Upper Extremity; GMFCS, Gross Motor Function Classification System; GMFM, Gross Motor Function Measure (Dimension D: standing, Dimension E: walking, running and jumping); MACS, Manual Ability Classification System; MA‑2, Motor Assessment Scale‑2; MAS, Modified Ashworth Scale; MF, Male/Female; NR, Not Reported; PBS, Pediatric Balance Scale; PDMS‑2, Peabody Developmental Motor Scales, Second Edition; PEDI, Pediatric Evaluation of Disability Inventory; PEDI‑CAT, Pediatric Evaluation of Disability Inventory Computerized Adaptive Test; PedsQoL, Pediatric Quality of Life Inventory; QUEST, Quality of Upper Extremity Skills Test; ROM, Range of Motion; TC, Topography of Cerebral Palsy; TUG, Timed Up and Go Test; WeeFIM, Functional Independence Measure for Children; 10MWT, 10‑Meter Walk Test; 6MWT, 6‑Minute Walk Test.

The experimental interventions comprised several clinically distinct categories of emerging rehabilitation technologies: robot-assisted rehabilitation, including RAGT (59, 69, 71, 78, 79, 82, 90, 92, 99, 101, 103), upper-limb and hand robotics (61, 72, 91, 98) and soft robotic exoskeletons (70); Gamified Rehabilitation (73, 96); VR rehabilitation, including immersive VR (64, 76) and non-immersive VR (58, 60, 62, 63, 65–68, 74, 77, 81, 83–89, 93–95, 97, 100, 102); metaverse-based physical therapy (75); and combined VR and robotic interventions (80). The duration of intervention for each trial is summarized in Table 2.

**Table 2:** Intervention characteristics and outcome measures of the studies included in the systematic review and meta-analysis.

Study	Emerging tech	Emerging technology model	Intervention	Session time (min)	Number of sessions	Frequency (ss/wk)	Duration of treatment (wk)	Qualitative findings
Tekeş et al. (59)	RAGT	Hocoma Lokomat^®^	CG PT EG PT + RAGT	60	15	2–3	5–7.5	EG significantly improved GMFM-D, GMFM-total score and GAS (*p* < 0.05, sustained 12 weeks); both groups improved GMFM-E (*p* < 0.05). No between-group differences in walking, spasticity or QoL (*p* > 0.05).
Julien et al. (69)	RAGT	G-EO system	CG PT EG RAGT	30/20	6/10	3/5	2	EG enhanced gait speed, 6MWT distance and PGI-I (*p* < 0.05) and increased sensorimotor network functional connectivity (*p* < 0.05). No significant changes in GMFM-D/E or corticospinal tract structural connectivity (*p* > 0.05).
Hui et al. (70)	Soft robotic exoskeleton	Relink-ANK	CG PT EG PT + SRE	60	40	5	8	Both groups improved (*p* < 0.01); EG showed greater improvements in all outcomes (*p* < 0.001)
Choi et al. (71)	RAGT	Angel Legs M20	CG PT EG PT + RAGT	30	18	3	6	EG greater improvements in GMFM-total, GMFM-E, and PEDI-CAT responsibility (*p* < 0.05) vs. CG. At 4-week follow-up, balance and GDI were further enhanced (*p* < 0.05).
Adar et al. (72)	Robotic upper limb training	AMADEO	CG PT EG PT + RUL	40	30	5	6	Both groups improved in hand function, dexterity, spasticity, and QoL (*p* < 0.05); no significant between-group differences in all outcomes (*p* > 0.05)
Yaśar et al. (78)	RAGT	RoboGait	CG PT EG PT + RAGT	65	16	2	8	Both groups improved in spasticity, balance, and functional outcomes (*p* < 0.05); no significant between-group differences in all measures (*p* > 0.05)
Moll et al. (79)	RAGT	Hybrid Assistive Limb	CG PT EG PT + RAGT	Inpatient / Inpatient + 20	6	6	11d	EG improved GMFM-total and D + E (*p* = 0.002; *p* = 0.046); no differences in 10MWT, 6MWT (*p* > 0.05)
Pool et al. (82)	RAGT	RT600	CG LT EG LT + RAGT	60	18	3	6	Both groups improved in all outcomes (*p* < 0.05); no significant between-group differences in GAS, 10mWT, WeeFIM, COPM, or GMFM (*p* > 0.05)
Wallard et al. (90)	RAGT	Lokomat^®^Pediatric	CG PT EG PT + RAGT	40	20	5	4	EG improved gait parameters, GMFM-D/E, and COM-COP kinetics (*p* < 0.05); no significant changes in controls (*p* > 0.05).
El-Shamy, (91)	Robotic upper limb training	Armeo^®^ Spring	CG PT EG PT + RUL	45	36	3	12	EG reduced MAS (*p* < 0.05) and increased QUEST total score (*p* < 0.05) versus CG.
Wu et al. (92)	RAGT	3DCaLT	CG PT EG PT + RAGT	30–40	18	3	6	EG showed significant improvements in walking speed and 6MWT (*p* = 0.03; *p* = 0.048); greater improvement in 6MWT compared with CG (*p* = 0.01); CG improved only in GMFM-D (*p* = 0.01)
Gilliaux et al. (98)	Robotic upper limb training	REAPlan	CG PT EG PT + RUL	45	40	5	8	EG improved movement smoothness (*p* < 0.01) and Box and Block score (*p* = 0.04); both groups gained QUEST dissociated movements (*p* < 0.05). No other changes.
Sarhan et al. (99)	RAGT	Lokomat^®^ Pro Version 4	CG PT EG PT + RAGT	30–40	30	3	10	EG showed significant improvements in stride length, cadence, gait velocity, and balance (all *p* < 0.001); CG improved only in balance (*p* < 0.005)
Druzbicki et al. (101)	RAGT	Hocoma Lokomat^®^	CG PT EG PT + RAGT	45	20	5	4	EG improved hip flexion ROM (*p* = 0.0065) and coronal plane pelvic motion (*p* < 0.05); no significant between-group differences in gait speed, step length, or cadence (*p* > 0.05)
Smania et al. (103)	RAGT	Gait Trainer GT I	CG PT EG PT + RAGT	40	10	5	2	EG showed significant improvements in 10MWT, 6MWT, gait speed, step length, and hip kinematics (all *p* < 0.05); effects maintained at 1-month follow-up; CG showed no significant changes (*p* > 0.05)
Peramalaiah et al. (61)	Robotic upper limb training	robotic manipulandum device	CG PT EG PT + RUL	45	24	3	8	Both groups improved in PDMS-2 and CUE outcomes (all *p* < 0.05); EG showed significantly greater gains in grasping, visual-motor integration, success rate, and movement error (all *p* < 0.05)
Karabulut et al. (68)	Non-immersive VR	Xbox 360 Kinect	CG PT EG PT + VR	45	16	2	8	EG improved ABILHAND-Kids, SCUES, shoulder/elbow proprioception (all *p* < 0.05); control improved only elbow flexion 60° (*p* = 0.001). Between-group differences in shoulder proprioception (*p* < 0.05).
Abd-Elfattah et al. (73)	Gamified Rehabilitation	iPad 3	CG PT EG PT + GAME	60	36	3	12	Both groups improved in QUEST, pinch strength, and Nine-Hole Peg Test (all *p* < 0.001); GAME showed significantly greater improvements in all outcomes (all *p* < 0.05)
Wang et al. (81)	Non-immersive VR	Nintendo Wii	CG CIT EG CIT + VR	135	16	2	8	Both groups improved in manual dexterity, bimanual function, and playfulness (all *p* < 0.05); CG showed greater gains in self-care (*p* < 0.05); parental stress decreased only in EG (*p* < 0.05); no between-group differences in motor outcomes (*p* > 0.05)
Jung et al. (83)	Non-immersive VR	Xbox 360 Kinect	CG PT EG PT + VR	40	18	3	6	EG improved SCALE (except right hip abduction) and PBS (all *p* < 0.05); no between-group gait differences (*p* > 0.05).
El-Shamy et al. (88)	Non-immersive VR	Nintendo Wii	CG PT EG PT + VR	60	36	3	12	EG reduced MAS, increased power grip, pinch grip and PDMS-2 hand function (all *p* < 0.05) vs. usual care.
Sajan et al. (93)	Non-immersive VR	Nintendo Wii	CG PT EG PT + VR	45	18	6	3	EG improved QUEST total (*p* = 0.027) and grasp (*p* = 0.039); both groups enhanced balance, walking, BBT (*p* < 0.05). No between-group differences (*p* > 0.05).
Tarakci et al. (94)	Non-immersive VR	Nintendo Wii-Fit	CG PT EG PT + VR	50	24	2	12	EG greater improvements in FRT, STS, TUG, 10MWT, 10SCT and WeeFIM versus conventional training (all *p* < 0.05).
Chiu et al. (100)	Non-immersive VR	Wii Sports Resort™	CG PT EG PT + VR	40	18	3	6	EG showed no significant improvements in coordination, grip strength, or hand function (all *p* > 0.05). Caregivers perceived increased hand use quantity at 12 weeks (*p* = 0.03).
Yenilmez et al. (58)	Non-immersive VR	Xbox 360 Kinect	CG PT EG PT + VR	45	24	2	12	EG showed significantly greater improvements in GMFM, balance (one-leg standing), and functional mobility (TUG) compared with CG (all *p* < 0.05); CG showed improvements only in partial indicators.
Roberts et al. (60)	Non-immersive VR	Nintendo Wii	CG CIMT EG CIMT+VR	360	10	5	2	Both groups improved in AHA and COPM scores (all *p* < 0.05); no significant between-group differences in upper limb function and occupational performance (*p* > 0.05); VR enhanced child engagement and acceptability
Dwarkadas et al. (62)	Non-immersive VR	ESP32 with IMU sensor module	CG PT EG PT + VR	90	24	3	8	EG showed greater improvements in PBS balance, intrinsic motivation (interest/enjoyment, perceived competence), coordination, and game performance compared with CG (all *p* < 0.05); EG also had reduced pressure/tension during training
Daliri et al. (63)	Non-immersive VR	Leap Motion Controller	CG OT EG OT + VR	45	12	2	6	EG showed greater reductions in spoon/knife use time and greater improvements in grip strength compared with CG (all *p* < 0.05); no significant between-group difference in wrist extension ROM (*p* > 0.05)
Baillet et al. (64)	Immersive VR	HTC Vive Pro Eye	CG PT + OT EG PT + OT + VR	45	12	3	4	EG showed significantly greater improvements in MABC-2 manual dexterity, elbow flexion/shoulder adduction ROM, movement accuracy, speed, and fluency compared with CG (all *p* < 0.05); both groups reported high motivation and no serious adverse events
Ziab et al. (65)	Non-immersive VR	Xbox 360 Kinect	CG PT EG1 VR EG2 BST	60	18	3	6	EG1 (VR) and EG2 (BST) showed significantly greater improvements in balance (PBS), gross motor function (GMFM D&E), and center of mass displacement compared with CG (all *p* < 0.05); no significant differences between EG1 and EG2 in most outcomes
Mouhamed et al. (66)	Non-immersive VR	Nintendo Wii Fit Plus with balance board	CG PT EG PT + VR	60	36	3	12	EG showed significantly greater improvements in Pediatric Balance Scale (PBS) scores and greater reductions in overall, anteroposterior, and mediolateral stability indices compared with CG (all *p* < 0.05)
Madboly et al. (67)	Non-immersive VR	Nintendo Wii-Fit	CG PT EG1 PT + VR EG2 PT + Balance beam	60	36	3	12	EG1 and EG2 showed significantly greater improvements in balance (HUMAC balance system) and 6MWT compared with CG (all *p* < 0.01); no significant differences between EG1 and EG2 (*p* > 0.05)
Saussez et al. (74)	Non-immersive VR	REAtouch	CG HABIT-ILE EG HABIT-ILE + VR	90–120	10–12	5–6	2	Both groups significantly improved in upper limb function, daily activities, and goal attainment (all *p* < 0.05); EG showed non-inferior efficacy compared with CG in all primary and secondary outcomes
Menekseoglu et al. (76)	Immersive VR	Oculus Quest 128GB	CG PT EG PT + VR	60	12	2	6	EG showed significantly greater improvements in AHA, ABILHAND-Kids, QUEST, KINDL, and active upper extremity ROM compared with CG (all *p* < 0.001); effects maintained at 3-month follow-up
Choi et al. (77)	Non-immersive VR	RAPAEL Smart Kids	CG OT EG OT + VR	30	30	5	6	EG showed significant improvements in shoulder function, movement efficiency and social cognition (*p* < 0.05). No between-group differences in upper extremity function. Feasible and high satisfaction.
Jha et al. (84)	Non-immersive VR	Xbox 360 Kinect	CG PT EG PT + VR	60	24	4	6	EG improved balance significantly more than CG (*p* < 0.05); similar effects on gross motor and daily function between groups
Choi et al. (85)	Non-immersive VR	RAPAEL Smart Kids	CG OT EG OT + VR	60	20	5	4	EG better in dexterity, ADL, forearm supination (p < 0.05); greater gains in severe impairment
Arnoni et al. (86)	Non-immersive VR	Xbox 360 Kinect	CG PT EG PT + VR	50	16	2	8	EG improved TUG, gait velocity, stride time, and pelvic motion (*p* < 0.05)
Sahin et al. (87)	Non-immersive VR	Xbox 360 Kinect	CG OT EG OT + VR	45	16	2	8	EG better in gross/fine motor and daily function (*p* < 0.001)
Okmen et al. (89)	Non-immersive VR	Sony PlayStation 2 EyeToy	CG PT + OT EG PT + OT + VR	60	12	3	4	EG significantly improved BFMF, GMFCS, FMS vs. CG (*p* < 0.05)
Ren et al. (95)	Non-immersive VR	Q4 Situational Interaction Training System	CG PT + OT EG VR + OT	40	60	5	12	EG better in fine motor, gross motor, balance, and spasticity (*p* < 0.05)
Cho et al. 2016 (97)	Non-immersive VR	Nintendo Wii Fit Plus	CG TT + PT EG VRTT + PT	60	24	3	8	EG better in gait, balance, muscle strength, GMFM (*p* < 0.05)
Chen et al. (102)	Non-immersive VR	Eloton SimCycle Virtual Cycling System	CG PT EG VR	40	36	3	12	EG improved knee strength & distal femur aBMD (*p* < 0.05)
Fu et al. (80)	VR-augmented robotic rehabilitation	VR + Lokomat^®^	CG PT EG VR + RAGT	50	48/48	4	12	EG better in gait, spasticity, GMFM, 6MWT; 30% weight loss optimal (*p* < 0.05)
Mitchell et al. (96)	Gamified Rehabilitation	Mitii	CG PT EG PT + GAME	30	120	6	20	EG improved functional strength and 6MWT (*p* < 0.001); no change in daily activity
Moon et al. (75)	Metaverse Rehabilitation	Metaverse Rehabilitation Application	CG: PT EG: PT + Metaverse	30	12	3	4	EG better in GMFM, cardiopulmonary function; lower COVID-19 risk (*p* < 0.05)

BST, Behavioral Skill Training; CIMT, Constraint-Induced Movement Therapy; CIT, Constraint-Induced Therapy; GAME, Gamified Rehabilitation; HABIT-ILE, Hand-Arm Bimanual Intensive Therapy Including Lower Extremities; LT, Locomotor Training; OT, Occupational Therapy; PT, Physiotherapy; QoL, Quality of Life; RAGT, Robot-Assisted Gait Training; RUL, Robotic Upper Limb Training; SRE, Soft Robotic Exoskeleton; ss, Sessions; TT, Treadmill Training; VR, Virtual Reality; VRTT, Virtual Reality Treadmill Training; wk, Weeks.

With respect to assessment time points, all studies reported outcomes immediately post-intervention. In addition, four trials included short-term follow-up assessments conducted within 6 weeks (69, 71, 85, 103), and 11 trials performed medium- to long-term follow-up evaluations at 6 weeks or beyond (59, 63–65, 74, 76, 77, 82, 84, 92, 100). Finally, 24 of the included studies reported receiving external funding (60–64, 69, 71, 74, 76, 77, 79–82, 85, 86, 92, 95, 96, 98–100, 102, 103).

Regarding economic and safety outcomes, no included trial reported a formal cost-effectiveness evaluation. Four trials reported generally mild adverse events: two cases of tibial skin abrasion and one withdrawal due to leg pain during RAGT (59); one case each of transient skin redness and presyncope during RAGT (79); headache in three children and nausea and dizziness in one child after immersive VR (76); and two cases of minor musculoskeletal pain during web-based Mitii training (96). No severe intervention-related adverse events were reported.

### 3.3 Risk of Bias in included studies

**Figure 2:**
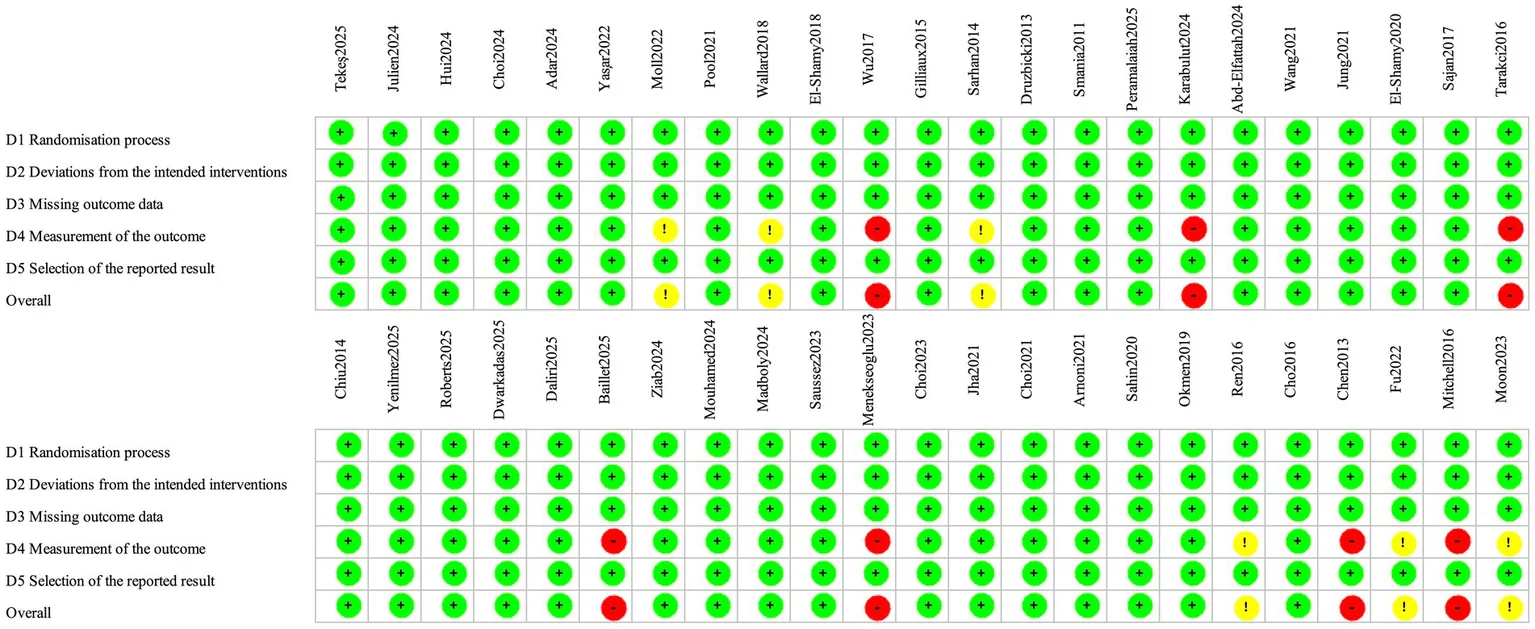
Risk of bias summary for individual studies.

**Figure 3:**
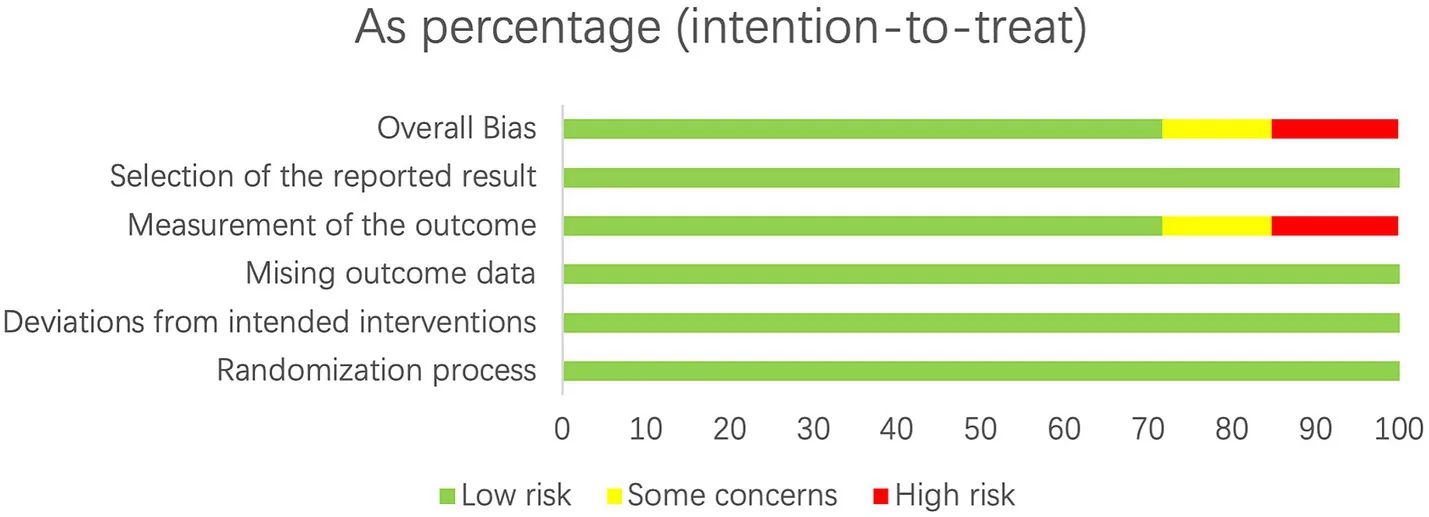
Risk of bias summary across methodological domains.

Risk of bias was assessed using the revised Cochrane Risk of Bias tool (RoB 2) across five domains (51). All 46 trials (100%) were judged to be at low risk of bias in the domains of the randomization process, deviations from intended interventions, missing outcome data, and selection of the reported result. Concerns were limited to the outcome measurement domain, in which 33 trials (71.7%) were rated as low risk, six (13.0%) as having some concerns, and seven (15.2%) as high risk. These concerns were primarily related to absent or unclear reporting of outcome-assessor blinding. Because all judgments in the remaining four domains were low risk, the overall risk-of-bias judgments mirrored those for the outcome measurement domain: 33 studies (71.7%) were classified as low risk, six (13.0%) as having some concerns, and seven (15.2%) as high risk. Detailed study-level assessments are presented in Figure 2, and the distribution of judgments across domains is summarized in Figure 3.

### 3.4 Quantitative synthesis

Quantitative synthesis was performed for 19 distinct outcome measures, spanning three principal functional domains: lower-extremity function, upper-extremity function, and global motor function. The primary pooled results are summarized in Table 3, and detailed effect estimates, heterogeneity statistics, and corresponding forest plots for key outcome measures are presented in the sections that follow.

**Table 3:** Summary of meta-analysis results comparing the emerging technology group with the conventional rehabilitation group.

Outcome measure	K	*N*	MD (95% CI)	*p*	Heterogeneity		Publication Bias	Trim and Fill	
					Q (df)	I^2^ (%) (*p*)	Egger *p*	Adj Effect Size	% var
Lower Limb Function									
10MWT (s)	6	172	−2.83 [−4.39, −1.27]	0.0004	2.95 (5)	0% (0.71)	—	—	—
6MWT (m)	10	414	43.56 [25.55, 61.57]	<0.00001	16.12 (9)	44% (0.06)	0.014	43.56 [25.55, 61.57]	0%
GMFM-Total (%)	8	291	2.22 [−0.62, 5.07]	0.13	6.28 (7)	0% (0.51)	—	—	—
GMFM-dimension D (%)	10	345	5.24 [1.53, 8.95]	0.006	27.71 (9)	68% (0.001*)	0.199	—	—
GMFM-dimension E (%)	10	345	6.66 [1.95, 11.38]	0.006	33.36 (9)	73% (0.0001*)	0.184	—	—
TUG (s)	3	78	−2.17[−3.60, −0.75]	0.003	3.96 (2)	50% (0.14)	—	—	—
PBS (points)	10	344	5.21 [4.06, 6.37]	<0.00001	7.23 (9)	0% (0.61)	0.480	—	—
MAS-Lower limb (grade)	4	121	−0.53 [−1.22, 0.17]	0.14	23.05 (3)	87% (<0.0001*)	—	—	—
WeeFIM Total-Lower limb (points)	6	210	6.71 [2.94, 10.49]	0.0005	1.83 (5)	0% (0.87)	—	—	—
WeeFIM Self-care (points)	3	130	3.22 [0.72, 5.73]	0.01	2.15 (2)	7% (0.34)	—	—	—
Upper Limb Function									
MAS-Upper limb (grade)	2	70	−0.40 [−0.59, −0.21]	<0.00001	0.00 (1)	0% (1.00)	—	—	—
QUEST-Total (%)	2	48	−1.80 [−18.77, 15.17]	0.84	5.76 (1)	83% (0.02*)	—	—	—
QUEST-Dissociated movement (%)	3	106	4.67 [0.99, 8.35]	0.01	14.87 (2)	87% (0.0006*)	—	—	—
QUEST-Grasp (%)	4	124	2.59 [−3.41, 8.58]	0.40	32.94 (3)	91% (<0.00001*)	—	—	—
ABILHAND-Kids (points)	5	121	2.00 [−1.01, 5.01]	0.19	2.23 (4)	0% (0.69)	—	—	—
BBT (blocks/min)	4	93	1.13 [−3.27, 5.53]	0.61	1.40 (3)	0% (0.71)	—	—	—
AHA (points)	2	70	−4.24 [−12.21, 3.74]	0.30	0.14 (1)	0% (0.71)	—	—	—
Global Motor Function									
WeeFIM Total (points)	7	229	6.67 [2.97, 10.37]	0.0004	1.84 (6)	0% (0.93)	—	—	—
COPM Performance (points)	3	110	0.14 [−0.37, 0.65]	0.60	1.49 (2)	0% (0.47)	—	—	—

% var., percentage of variation; 95% CI, 95% confidence interval; Adj, adjusted; df, degree of freedom; I^2^, degree of inconsistency; K, number of comparisons; MD, mean difference; *N*, total sample size; Ns, participants per study; *p*, *p*-value; Q, Q-test. *Indicates statistically significant between-study heterogeneity (Cochran’s Q test *p* < 0.05).

#### 3.4.1 Lower limb function

##### 3.4.1.1 10MWT

**Figure 4:**
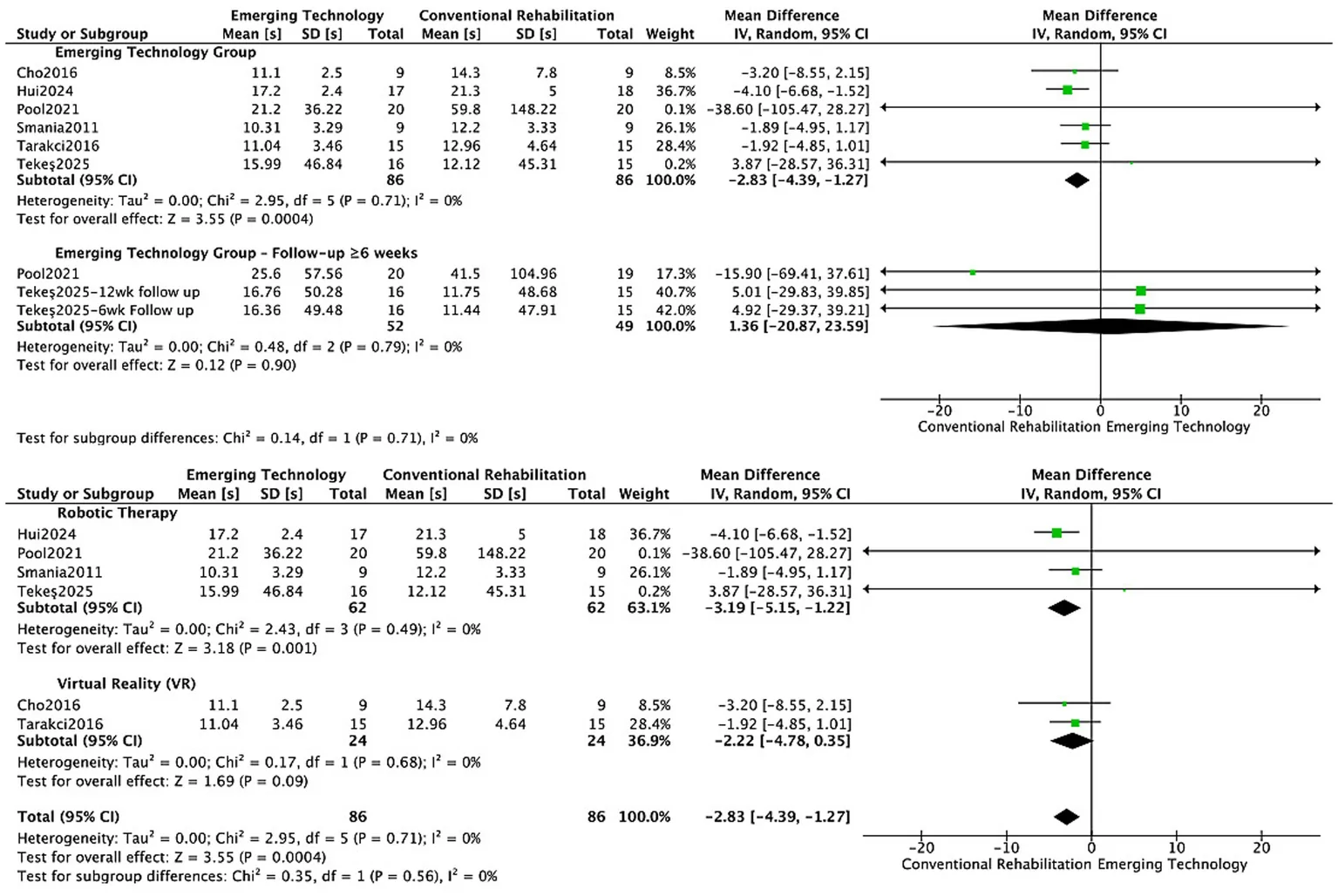
Forest plot of the meta-analysis for 10MWT.

Six studies involving 172 participants contributed data to the meta-analysis of 10MWT performance (Figure 4) (59, 70, 82, 94, 97, 103). Emerging technology interventions significantly reduced walking time compared with conventional rehabilitation (MD = −2.83, 95% CI −4.39 to −1.27, *p* = 0.0004). Robot-assisted interventions accounted for the greater share of this benefit (MD = −3.19, 95% CI −5.15 to −1.22, *p* = 0.001), whereas VR interventions showed only a non-significant trend toward improvement (MD = −2.22, 95% CI −4.78 to 0.35, *p* = 0.09), with no significant difference between the two modalities (Chi^2^ = 0.35, df = 1, *p* = 0.56, I^2^ = 0%). With respect to durability, the improvement was largely confined to the immediate post-intervention assessment, as the effect was no longer evident at follow-up ≥6 weeks (MD = 1.36, 95% CI −20.87 to 23.59, *p* = 0.90; test for subgroup differences: Chi^2^ = 0.14, df = 1, *p* = 0.71, I^2^ = 0%), although definitive conclusions regarding longer-term persistence are precluded by the limited number of studies with extended follow-up.

##### 3.4.1.2 6MWT

**Figure 5:**
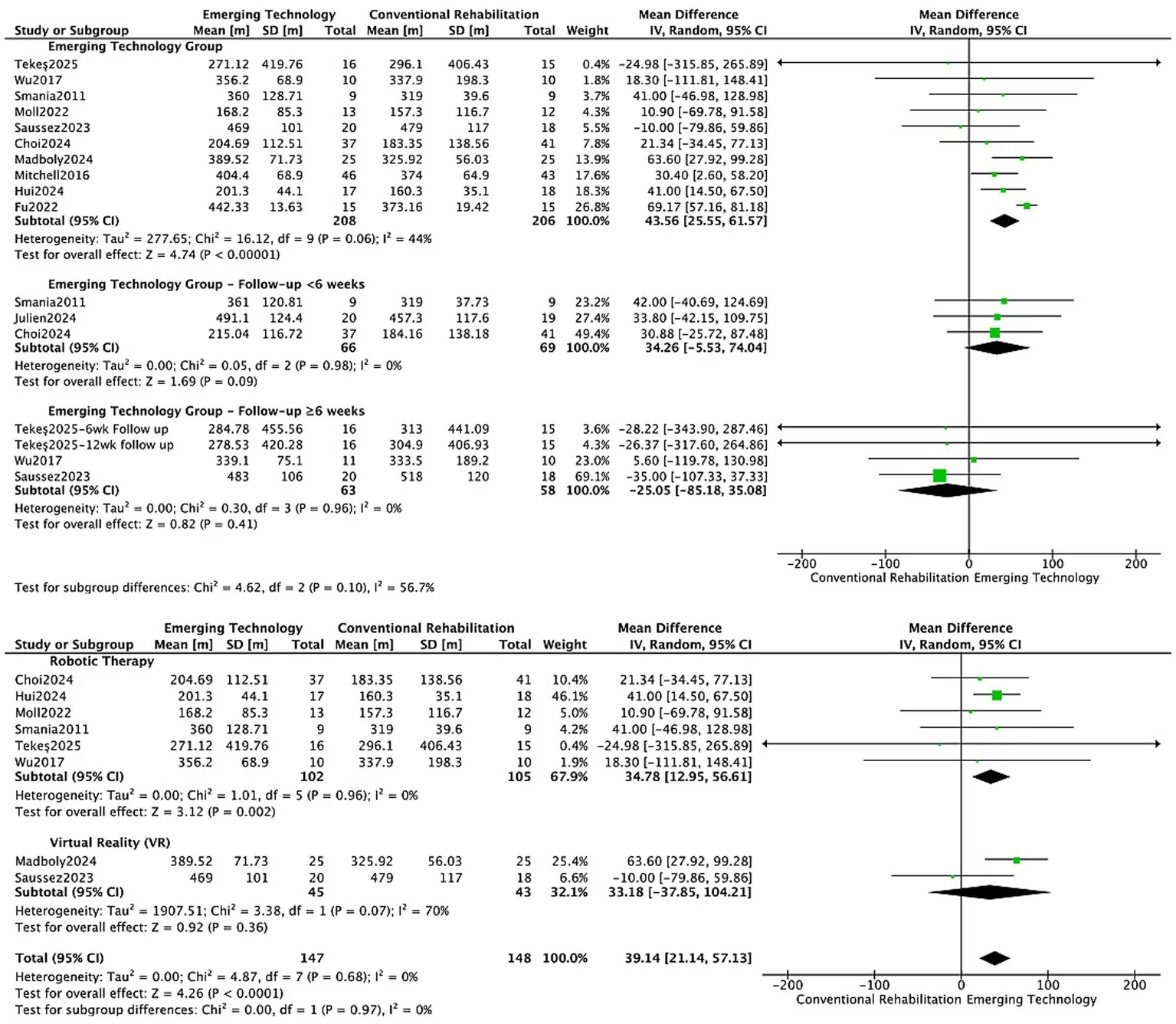
Forest plot of the meta-analysis for 6MWT.

Ten studies involving 414 participants contributed data to the meta-analysis of 6MWT performance (Figure 5) (59, 67, 70, 71, 74, 79, 80, 92, 96, 103). Emerging technology interventions significantly increased 6MWT walking distance compared with conventional rehabilitation (MD = 43.56, 95% CI 25.55 to 61.57, *p* < 0.00001, I^2^ = 44%). This overall benefit was driven primarily by robot-assisted interventions, which produced a significant improvement (MD = 34.78, 95% CI 12.95 to 56.61, *p* = 0.002, I^2^ = 0%), whereas VR interventions did not demonstrate a statistically significant effect and exhibited substantial heterogeneity (MD = 33.18, 95% CI − 37.85 to 104.21, *p* = 0.36, I^2^ = 70%); the difference between the two intervention types was not statistically significant (Chi^2^ = 0.00, df = 1, *p* = 0.97, I^2^ = 0%). The observed improvements were, however, largely confined to the immediate post-intervention period, as the effect was substantially attenuated at follow-up ≥6 weeks (MD = −25.05, 95% CI − 85.18 to 35.08, *p* = 0.41, I^2^ = 0%), with the test for subgroup differences by follow-up duration approaching but not reaching statistical significance (Chi^2^ = 4.62, df = 2, *p* = 0.10, I^2^ = 56.7%).

##### 3.4.1.3 Gross motor function

**Figure 6:**
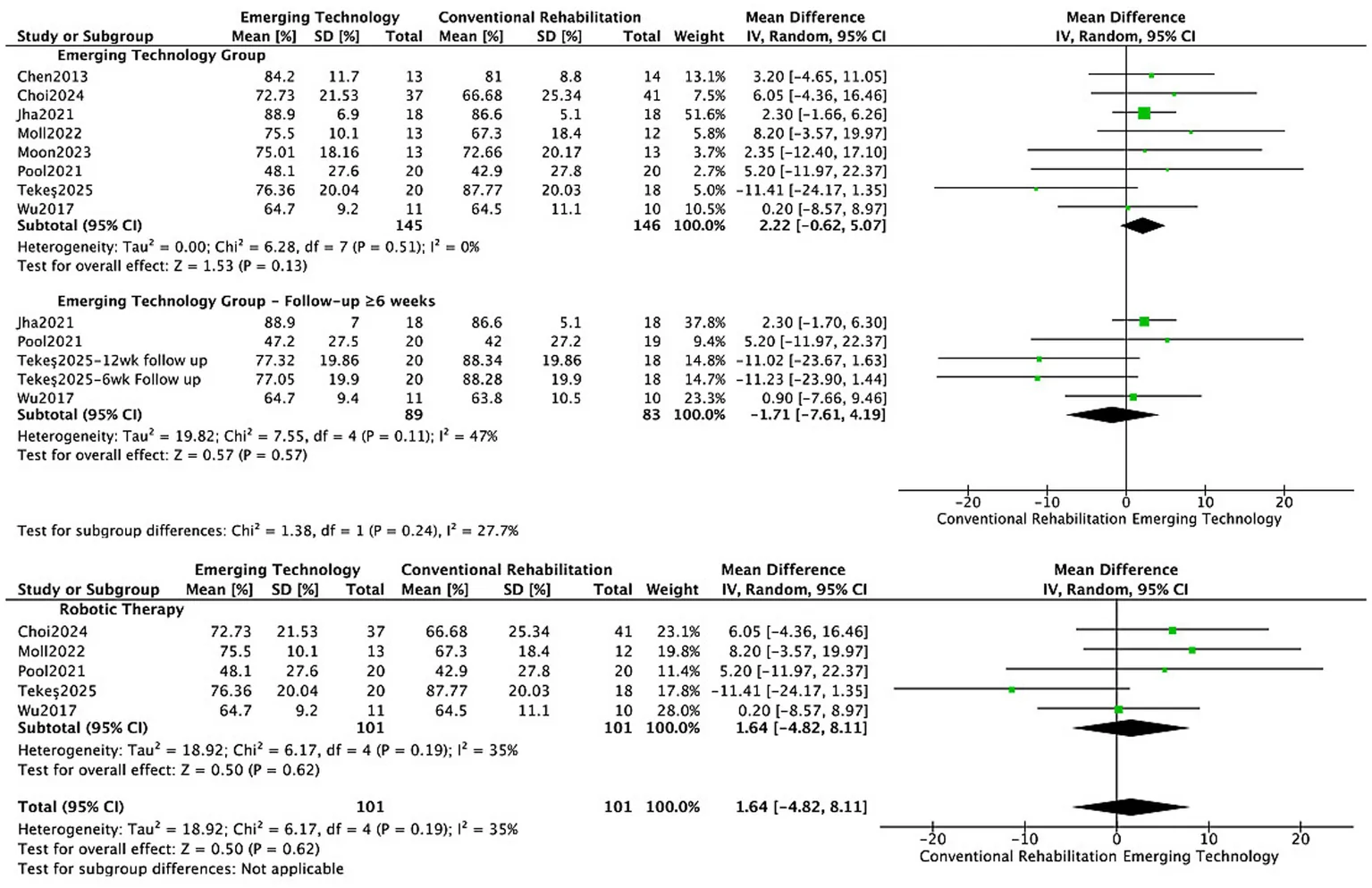
Forest plot of the meta-analysis for GMFM total score.

**Figure 7:**
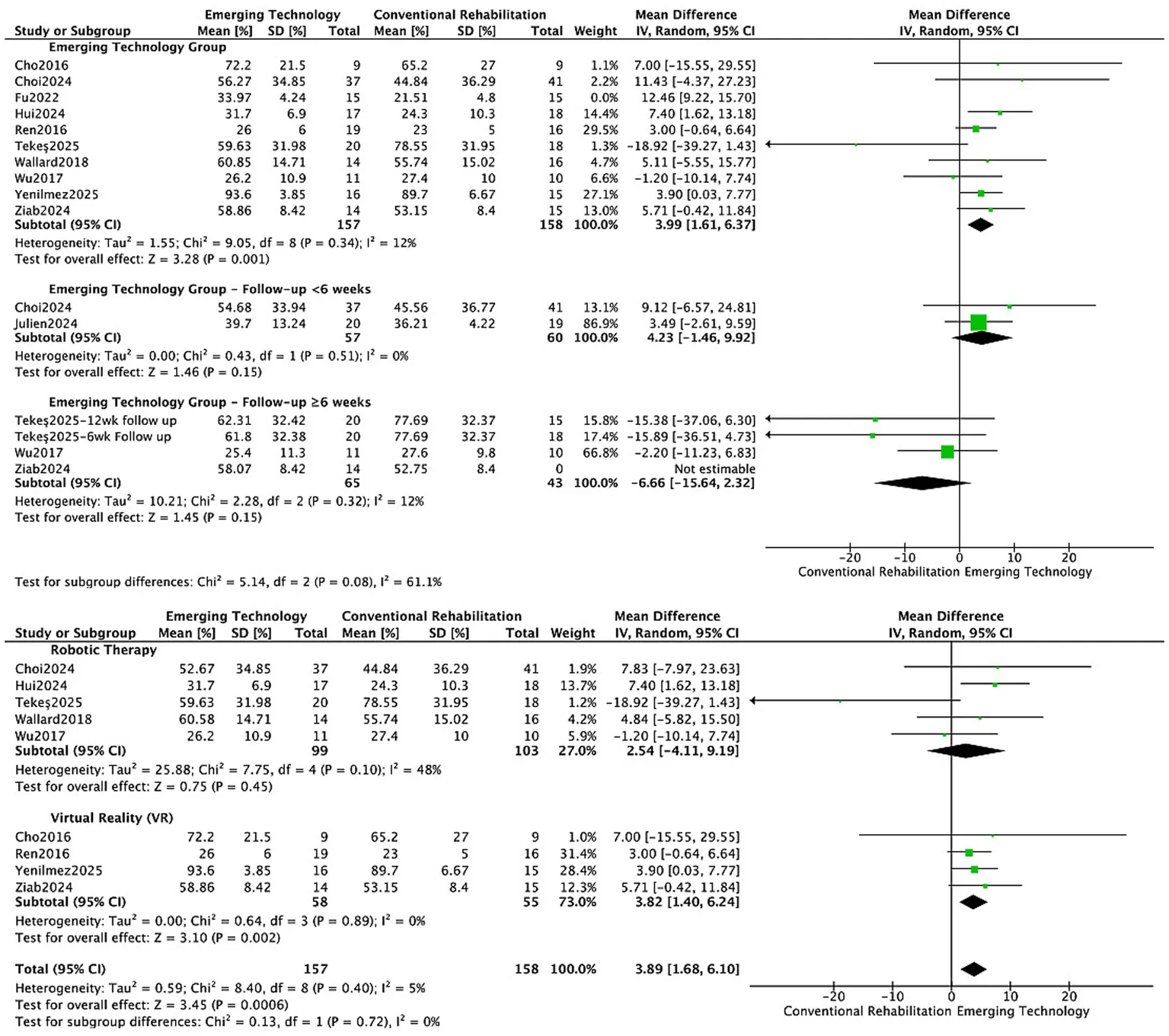
Forest plot of the meta-analysis for GMFM dimension D score.

**Figure 8:**
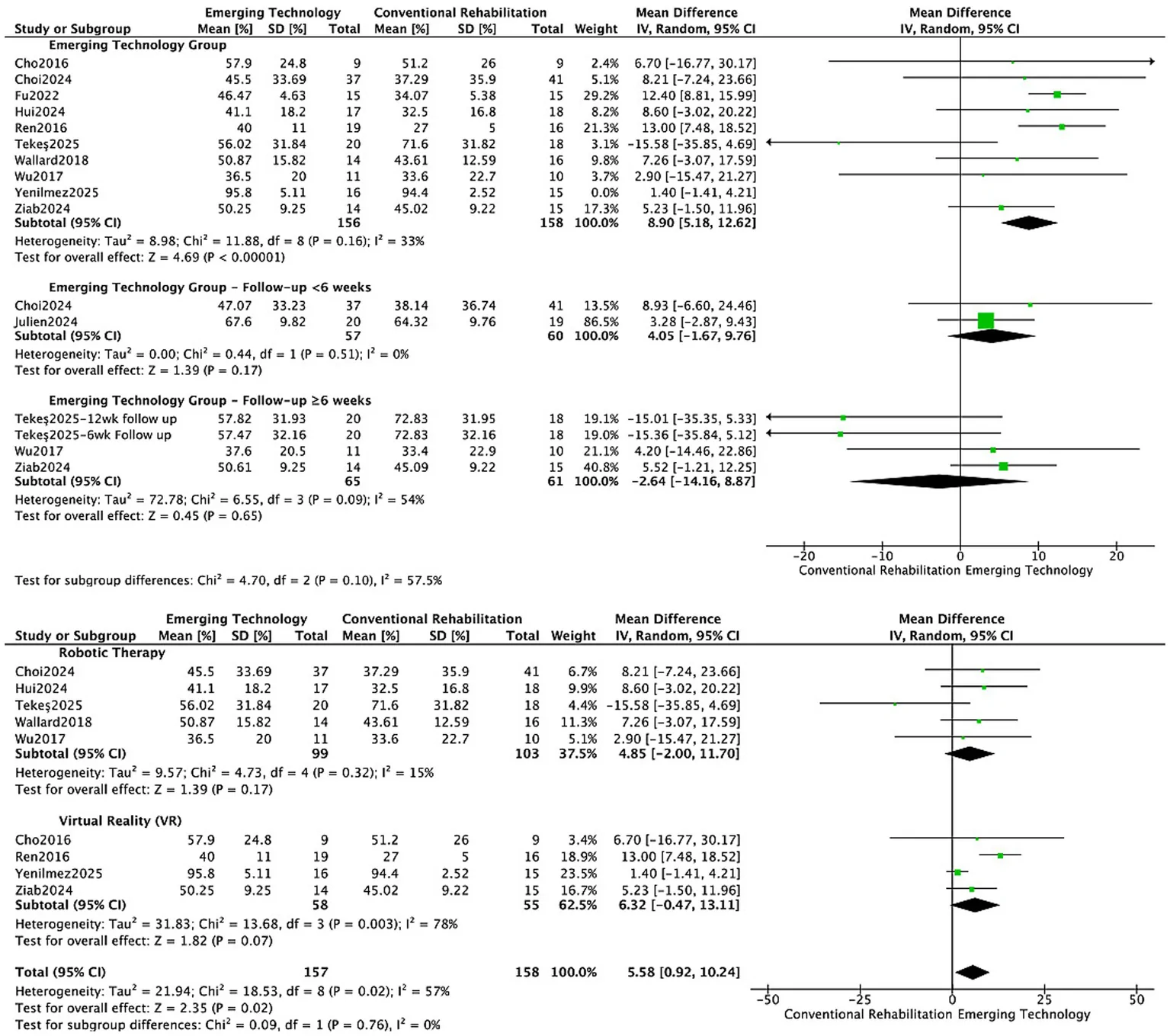
Forest plot of the meta-analysis for GMFM dimension E score.

Meta-analyses were performed for gross motor function as assessed by GMFM. Eight studies involving 291 participants contributed data for total GMFM score (Figure 6) (59, 71, 75, 79, 82, 84, 92, 102), 10 studies involving 345 participants for Dimension D (Standing; Figure 7) (58, 59, 65, 70, 71, 80, 90, 92, 95, 97), and 10 studies involving 345 participants for Dimension E (Walking, Running, and Jumping; Figure 8) (58, 59, 65, 70, 71, 80, 90, 92, 95, 97). Higher scores indicate better gross motor function. Emerging technology interventions significantly improved GMFM Dimension D (MD = 5.24, 95% CI 1.53 to 8.95, *p* = 0.006; I^2^ = 68%) and Dimension E (MD = 6.66, 95% CI 1.95 to 11.38, *p* = 0.006; I^2^ = 73%), whereas no significant improvement was observed in the GMFM total score (MD = 2.22, 95% CI −0.62 to 5.07, *p* = 0.13; I^2^ = 0%). Leave-one-out sensitivity analysis indicated that Fu 2022 was the main contributor to heterogeneity in the Dimension D analysis. Excluding this study reduced I^2^ from 68 to 12%, and the recalculated pooled estimate remained statistically significant (MD = 3.99, 95% CI 1.61 to 6.37, *p* = 0.001). For Dimension E, excluding Yenilmez 2025 reduced I^2^ from 73 to 33%, while the pooled effect also remained statistically significant (MD = 8.90, 95% CI 5.18 to 12.62, *p* < 0.00001). Thus, the sensitivity analyses substantially reduced heterogeneity without changing the statistical significance or direction of either pooled effect.

Across intervention types, VR was the only modality associated with a significant gain, specifically on Dimension D (MD = 3.82, 95% CI 1.40 to 6.24, *p* = 0.002, I^2^ = 0%), whereas robot-assisted interventions did not reach significance on any GMFM outcome. Subgroup differences between intervention types were not statistically significant (all *p* > 0.05).

These initial benefits faded considerably over time. At follow-up ≥6 weeks, improvements on Dimension D (MD = −6.66, 95% CI −15.64 to 2.32, *p* = 0.15) and Dimension E (MD = −2.64, 95% CI −14.16 to 8.87, *p* = 0.65) were no longer detectable, with the decline in Dimension D reaching significance in the subgroup comparison after exclusion of the influential trial (Chi^2^ = 5.81, df = 2, *p* = 0.05, I^2^ = 65.6%). These findings indicate that emerging technologies improve standing and walking-running-jumping function in the short term, with limited evidence that these gains persist beyond the immediate post-intervention period.

##### 3.4.1.4 TUG

**Figure 9:**
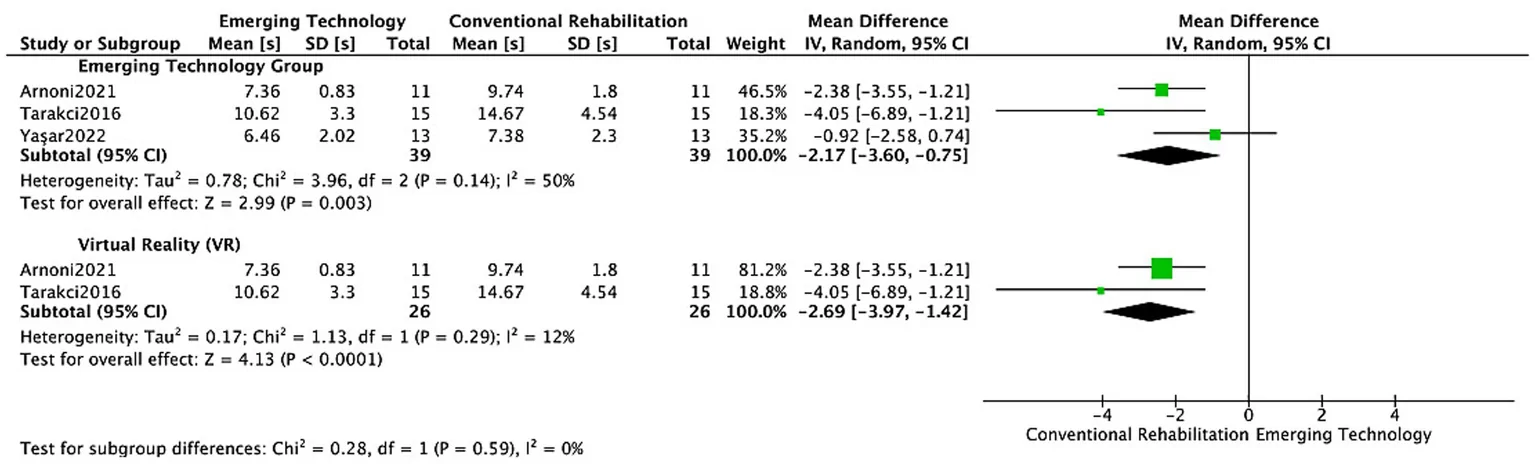
Forest plot of the meta-analysis for TUG.

Three studies involving 78 participants contributed data to the meta-analysis of TUG performance (Figure 9) (78, 86, 94). Emerging technology interventions significantly reduced TUG time compared with conventional rehabilitation (MD = −2.17, 95% CI −3.60 to −0.75, *p* = 0.003, I^2^ = 50%). VR interventions similarly yielded a significant benefit (MD = −2.69, 95% CI −3.97 to −1.42, *p* < 0.0001, I^2^ = 12%).

##### 3.4.1.5 PBS

**Figure 10:**
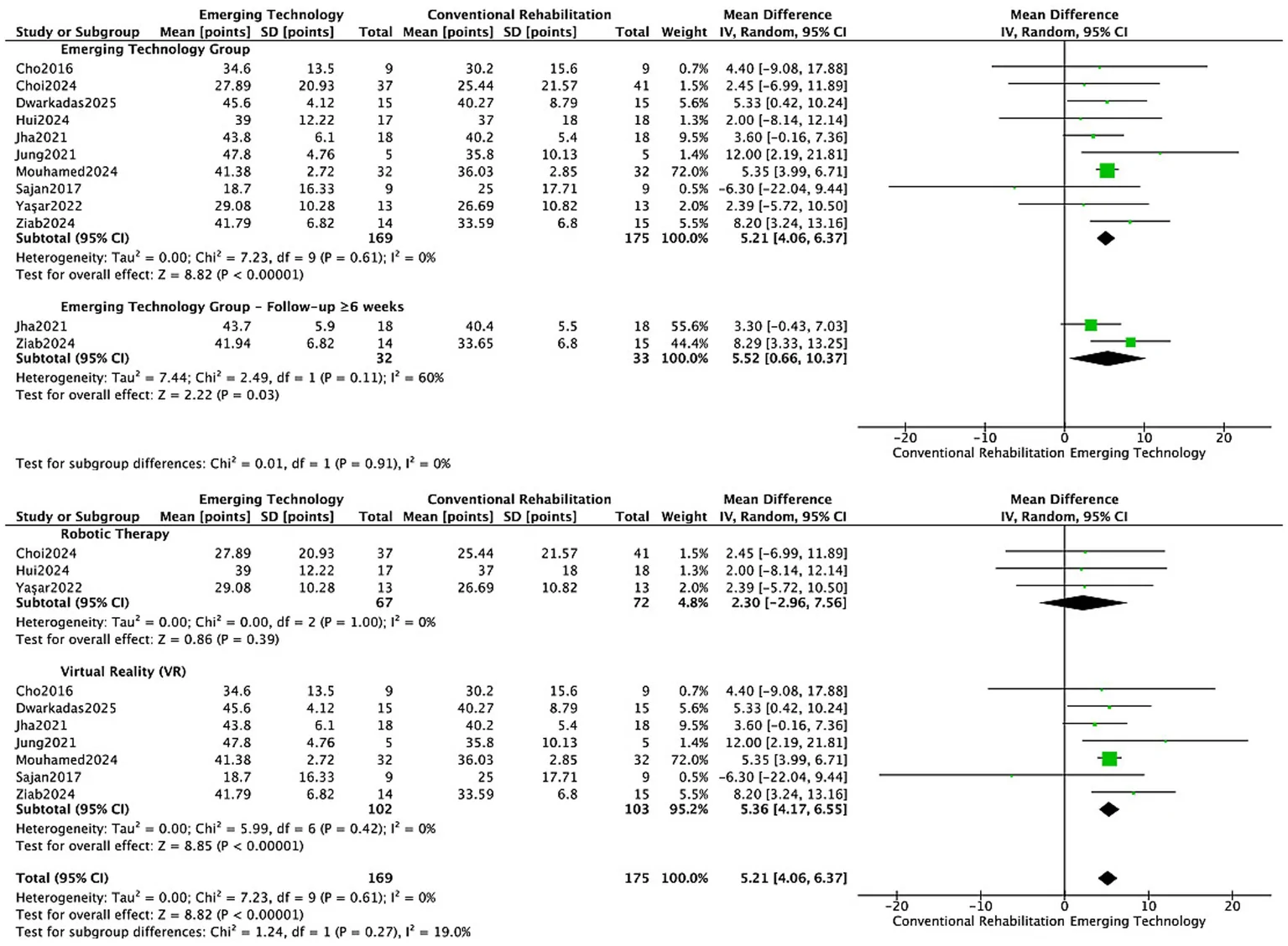
Forest plot of the meta-analysis for PBS.

Ten studies involving 344 participants contributed data to the meta-analysis of PBS performance (Figure 10) (62, 65, 66, 70, 71, 78, 83, 84, 93, 97). Emerging technology interventions significantly improved PBS scores compared with conventional rehabilitation (MD = 5.21, 95% CI 4.06 to 6.37, *p* < 0.00001, I^2^ = 0%). This benefit was particularly pronounced for VR interventions (MD = 5.36, 95% CI 4.17 to 6.55, *p* < 0.00001, I^2^ = 0%), whereas robot-assisted therapy did not yield a statistically significant effect (MD = 2.30, 95% CI −2.96 to 7.56, *p* = 0.39, I^2^ = 0%), with no significant difference detected between the two modalities (Chi^2^ = 1.25, df = 3, *p* = 0.74, I^2^ = 0%). Notably, significant benefits persisted at follow-up ≥6 weeks (MD = 5.52, 95% CI 0.66 to 10.37, *p* = 0.03, I^2^ = 60%), suggesting that gains in postural control may be more durable than those observed for gait or gross motor function.

##### 3.4.1.6 MAS-Lower limb

**Figure 11:**
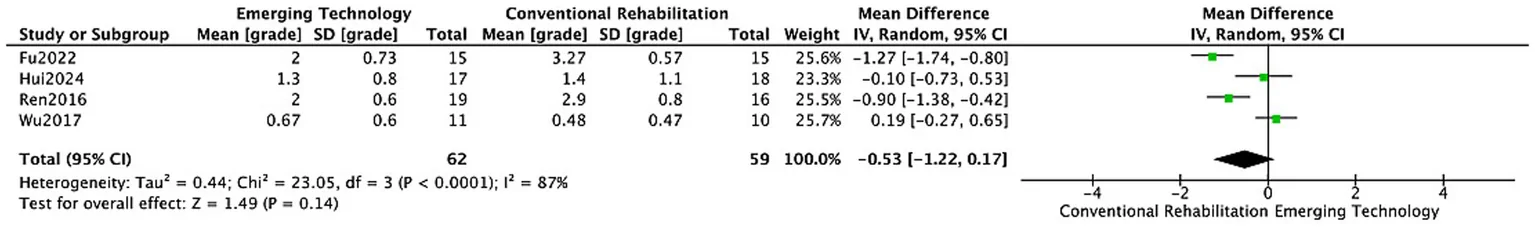
Forest plot of the meta-analysis for the lower limb MAS.

Four studies involving 121 participants contributed data to the meta-analysis of MAS lower-extremity score (Figure 11) (70, 80, 92, 95). Emerging technology interventions did not demonstrate a statistically significant difference compared with conventional rehabilitation (MD = −0.53, 95% CI −1.22 to 0.17, *p* = 0.14), with substantial between-study heterogeneity (I^2^ = 87%). Sensitivity analyses identified no single study as the dominant source of heterogeneity, suggesting that the observed variability likely reflects clinical differences in intervention protocols, participant characteristics, or outcome measurement across the included trials.

##### 3.4.1.7 WeeFIM total-lower limb

**Figure 12:**
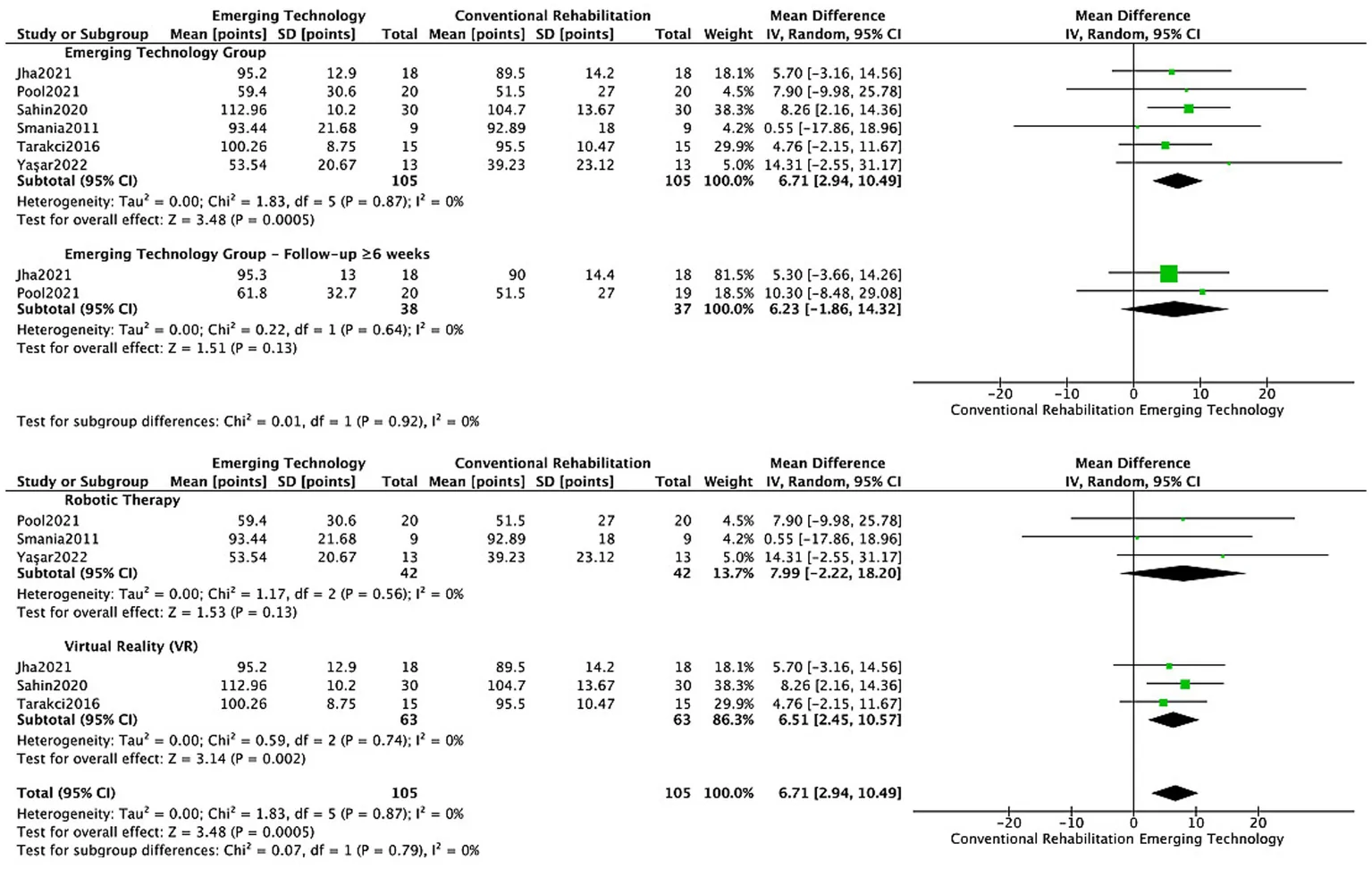
Forest plot of the meta-analysis for the lower limb WeeFIM total score.

Six studies involving 210 participants contributed data to the meta-analysis of WeeFIM lower extremity total score (Figure 12) (78, 82, 84, 87, 94, 103). Emerging technology interventions significantly improved this outcome compared with conventional rehabilitation (MD = 6.71, 95% CI 2.94 to 10.49, *p* = 0.0005, I^2^ = 0%). The gain was primarily driven by VR interventions (MD = 6.51, 95% CI 2.45 to 10.57, *p* = 0.002, I^2^ = 0%), whereas robot-assisted therapy did not reach statistical significance (MD = 7.99, 95% CI −2.22 to 18.20, *p* = 0.13, I^2^ = 0%), with no significant difference detected between the two modalities (Chi^2^ = 0.07, df = 1, *p* = 0.79, I^2^ = 0%). At follow-up ≥6 weeks, the benefit was attenuated and no longer statistically significant (MD = 6.23, 95% CI −1.86 to 14.32, *p* = 0.13, I^2^ = 0%), suggesting limited durability of lower-extremity functional gains beyond the immediate post-intervention period.

##### 3.4.1.8 WeeFIM self-care-lower limb

**Figure 13:**
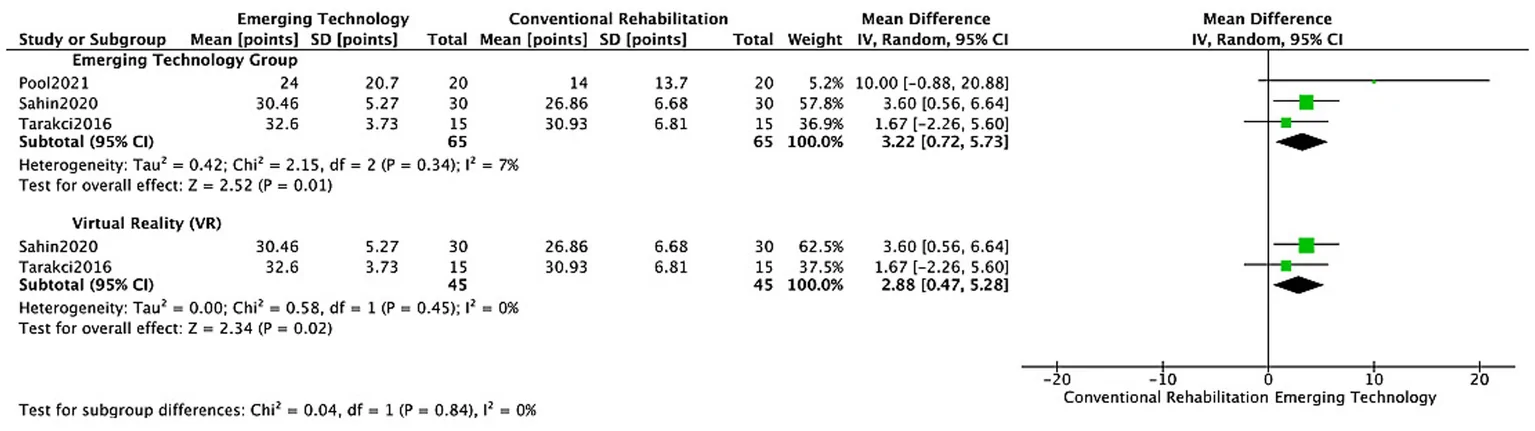
Forest plot of the meta-analysis for the lower limb WeeFIM Self-care subscale.

Three studies involving 130 participants contributed data to the meta-analysis of WeeFIM Self-Care subscale (Figure 13) (82, 87, 94). Emerging technology interventions significantly improved this outcome compared with conventional rehabilitation (MD = 3.22, 95% CI 0.72 to 5.73, *p* = 0.01, I^2^ = 7%). VR interventions yielded a significant benefit (MD = 2.88, 95% CI 0.47 to 5.28, *p* = 0.02, I^2^ = 0%), with no significant difference detected between intervention types (Chi^2^ = 0.04, df = 1, *p* = 0.84, I^2^ = 0%).

#### 3.4.2 Upper limb function

##### 3.4.2.1 MAS-upper limb

**Figure 14:**
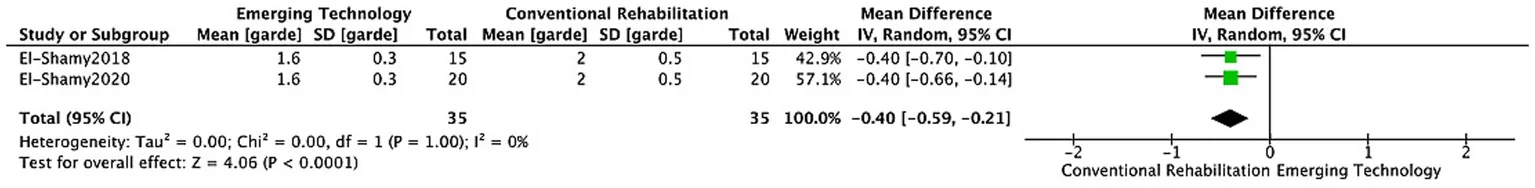
Forest plot of the meta-analysis for the upper limb MAS.

Two studies involving 70 participants contributed data to the meta-analysis of MAS upper-extremity score (Figure 14) (88, 91). Emerging technology interventions significantly reduced MAS-upper limb score compared with conventional rehabilitation (MD = −0.40, 95% CI − 0.59 to −0.21, *p* < 0.0001, I^2^ = 0%).

##### 3.4.2.2 QUEST

**Figure 15:**
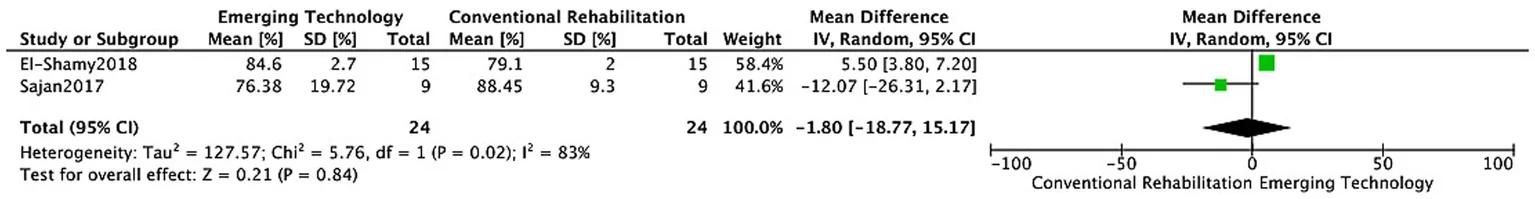
Forest plot of meta-analysis for QUEST total score.

Meta-analyses were performed for QUEST, with higher scores indicating superior fine motor function. Two studies involving 48 participants contributed data for QUEST Total score (Figure 15) (88, 91), three studies involving 106 participants for Dissociated Movement (Supplementary Figure S1) (73, 91, 98), and four studies involving 124 participants for Grasp subscore (Supplementary Figure S2) (73, 91, 93, 98). Emerging technology interventions significantly improved Dissociated Movement scores compared with conventional rehabilitation (MD = 4.67, 95% CI 0.99 to 8.35, *p* = 0.01, I^2^ = 87%). No statistically significant differences were detected for QUEST Total score (MD = −1.80, 95% CI −18.77 to 15.17, *p* = 0.84, I^2^ = 83%) or Grasp subscore (MD = 2.59, 95% CI −3.41 to 8.58, *p* = 0.40, I^2^ = 91%). Sensitivity analyses identified El-Shamy 2018 as the primary source of heterogeneity for both Dissociated Movement (I^2^ reduced from 87 to 0% upon exclusion) and Grasp subscore (I^2^ reduced from 91 to 0%), without altering the conclusions for either outcome.

##### 3.4.2.3 ABILHAND-Kids

Five studies involving 121 participants contributed data to the meta-analysis of ABILHAND-Kids performance (Supplementary Figure S3) (68, 72, 74, 81, 98). Emerging technology interventions did not significantly improve ABILHAND-Kids scores compared with conventional rehabilitation (MD = 2.00, 95% CI −1.01 to 5.01, *p* = 0.19, I^2^ = 0%). Neither robot-assisted therapy (MD = 3.10, 95% CI −5.33 to 11.52, *p* = 0.47, I^2^ = 0%) nor VR interventions (MD = −0.58, 95% CI −5.65 to 4.48, *p* = 0.82, I^2^ = 0%) demonstrated a significant effect. However, a significant improvement emerged in the ≥6 weeks follow-up subgroup (MD = 2.54, 95% CI 0.41 to 4.66, *p* = 0.02, I^2^ = 0%), suggesting a possible delayed benefit in hand function that was not apparent immediately post-intervention. The test for subgroup differences was not statistically significant (Chi^2^ = 1.30, df = 3, *p* = 0.73, I^2^ = 0%).

#### 3.4.3 Global motor function

Seven studies involving 229 participants contributed data to the meta-analysis of WeeFIM total score (Supplementary Figure S4) (72, 78, 82, 84, 87, 94, 103). Emerging technology interventions significantly improved WeeFIM total score compared with conventional rehabilitation (MD = 6.67, 95% CI 2.97 to 10.37, *p* = 0.0004, I^2^ = 0%).

In addition, no statistically significant differences between emerging technology interventions and conventional rehabilitation were observed for several secondary outcome measures, including BBT, AHA, and COPM, which reflects overall life satisfaction. Detailed results for these outcomes are presented in Table 3.

### 3.5 Publication Bias

Publication bias was assessed using funnel plots and Egger’s regression test for outcomes with 10 or more studies. Egger’s test suggested potential publication bias for 6MWT (*p* = 0.014). A subsequent trim-and-fill analysis, however, did not impute any missing studies and returned the original pooled estimate (MD = 43.56, 95% CI 25.55 to 61.57), indicating that the potential small-study effects do not materially influence the overall finding. For the remaining outcomes, no significant small-study effects were detected (GMFM Dimension D: *p* = 0.199; GMFM Dimension E: *p* = 0.184; PBS: *p* = 0.480; Appendix S2).

### 3.6 Sensitivity analysis

Sensitivity analyses using a leave-one-out approach were performed for all outcomes with substantial heterogeneity (I^2^ > 50%). For most outcomes, the iterative removal of any single study did not materially alter the direction or statistical significance of the pooled estimates. For GMFM Dimension D, removing Fu et al. (80) reduced heterogeneity from I^2^ = 68 to 12%, while the pooled MD remained statistically significant (*p* = 0.001). For GMFM Dimension E, removing Yenilmez et al. (58) reduced heterogeneity from I^2^ = 73 to 33%, with the pooled estimate remaining highly significant (*p* < 0.00001). For TUG, removing Yasar et al. (78) reduced heterogeneity from I^2^ = 50 to 12%, with the pooled estimate remaining highly significant (*p* < 0.0001). For QUEST Total (I^2^ = 83%), leave-one-out analysis could not be implemented, as only two studies provided extractable data. For QUEST Dissociated Movement and QUEST Grasp., removing El-Shamy (91) reduced I^2^ values from 87 and 91 to 0%, respectively, without altering the conclusions for either outcome. For MAS-Lower Limb (I^2^ = 87%), no single study was identified as the dominant source of heterogeneity, and the observed variability is likely attributable to clinical discrepancies across the included trials. These analyses confirm that the overall findings are robust and not unduly driven by any individual trial.

### 3.7 Certainty of evidence

We evaluated the certainty of evidence via the GRADE framework, and constructed the summary of findings tables using GRADEpro Guideline Development Tool (GRADEpro GDT). Detailed outcome-specific assessments and explanations for downgrading are presented in Appendix S3. No outcomes were rated as high certainty; 11 outcomes were rated as moderate certainty (10MWT, 6MWT, PBS, WeeFIM Total-Lower limb, WeeFIM Self-care-Lower limb, MAS-Upper limb, ABILHAND-Kids, BBT, AHA, WeeFIM Total, COPM Performance); six as low certainty (GMFM-Total, GMFM Dimension D, GMFM Dimension E, TUG, QUEST-Dissociated movement, QUEST-Grasp); and two as very low certainty (MAS-Lower limb and QUEST-Total). Imprecision was the most frequent reason for downgrading, affecting 18 outcomes, primarily because the individual pooled sample sizes fell below the optimal information size (OIS), resulting in wide 95% CIs. Additionally, seven outcomes were further downgraded due to substantial inconsistency across trials.

## 4 Discussion

Children with CP exhibit central motor and postural dysfunction, which compromises gross motor development, gait, balance, and independence in daily living (104). Conventional rehabilitation centered on physical therapy confers modest improvements, but limitations in treatment intensity, patient engagement, and long-term adherence may restrict therapeutic durability (105). Emerging technologies, including RAGT, VR, and game-based rehabilitation, offer high-intensity, repetitive practice with enhanced interactivity and multisensory feedback. This meta-analysis synthesizes RCT evidence to inform clinical decision-making for pediatric CP rehabilitation.

Prior meta-analyses have largely been confined to single modalities. Cortés-Pérez et al. (106) and AlSoqih et al. (34) reported potential benefits of robot-assisted and VR interventions, while Perez Carcamo et al. (37) and Bajpai et al. (107) extended these findings to non-immersive VR and active video games. However, these syntheses share important limitations: a predominant focus on short-term lower-extremity outcomes and an absence of integrative cross-technology comparisons. The present review addresses these gaps by encompassing 46 trials and 1,812 children, spanning multiple technologies across lower-extremity, upper-extremity, and global functional domains. It also compares immediate post-intervention outcomes, short-term follow-up outcomes, and medium- to long-term follow-up outcomes, suggesting that effect patterns vary across technologies and supporting nuanced, technology-specific clinical interpretation.

The evidence base synthesized in this review was predominantly concentrated on RAGT and non-immersive VR, whereas RCTs evaluating immersive VR, soft robotic exoskeletons, gamified rehabilitation, and metaverse-based interventions remained comparatively sparse. This uneven distribution of research across technological modalities limits robust cross-technology comparisons and highlights the need for further clinical evaluation of less-studied approaches.

Consistent with prior work, emerging technologies significantly improved gait, balance, gross motor function, and functional independence. Robot-assisted interventions showed significant benefits mainly for walking speed and endurance, whereas VR interventions showed significant benefits mainly for balance and standing function. Lower-extremity spasticity showed no significant improvement. Regarding upper-extremity function, emerging technologies reduced spasticity and improved dissociated movement, but benefits for overall upper-extremity function and grasp were limited. Improvements measured using ABILHAND-Kids were detected at follow-up of ≥6 weeks but not immediately after intervention. No significant between-group differences were observed for BBT, AHA, or COPM.

Analyses stratified by follow-up duration showed that significant motor benefits were detected mainly immediately after intervention. At follow-up of ≥6 weeks, significant effects were detected only for balance and hand function. This pattern may partly reflect the limited number of studies with extended follow-up and should not be interpreted as definitive evidence that other treatment effects were not maintained. These findings highlight the need to evaluate maintenance strategies and include longer follow-up periods in future trials.

The rehabilitative benefits of emerging technologies may be explained by principles of motor learning and neuroplasticity (108). Task-specific and repetitive training may facilitate motor-memory consolidation and neuroplastic adaptation within motor-related neural networks (109–111). Structured training with these technologies may also increase physical activity and sustain effective engagement in rehabilitation tasks (112). These findings indicate that the benefits of emerging technologies are multidimensional but vary in durability, with lower-extremity and global functional gains evident primarily in the short term and hand function improvements being detected at later follow-up. Given this observation, future protocols should prioritize precisely targeted upper-extremity training that leverages intelligent rehabilitation devices to optimize fine motor outcomes, with prolonged monitoring to capture late-emerging benefits.

## 5 Clinical implications

Interpretation of pooled effect estimates in relation to minimal clinically important differences (MCIDs) provides additional insight into their potential clinical relevance. These comparisons should, however, be regarded as supportive rather than definitive, because published MCID estimates are population- and method-specific and may not correspond directly to pooled between-group effects (113). For the 6MWT, the pooled improvement of 43.56 m exceeds published MCID estimates of 6–23 m overall and 4–28 m for children at GMFCS levels I–II, suggesting a potentially clinically meaningful group-level improvement in walking endurance (114). For the 10MWT, the pooled reduction of 2.83 s indicates improved walking performance; however, available MCID values are expressed in m/s rather than seconds, precluding direct comparison (115). For the TUG, the pooled reduction of 2.17 s is within the broad range of published MCID estimates of approximately 0.22–5.31 s, although these estimates vary by GMFCS level and estimation method (116). For the PBS, the 5.21-point improvement lies within the reported MCID range of 3.66–5.83 points, indicating potential clinical relevance (117). Overall, these findings support the potential clinical value of technology-assisted rehabilitation as a component of individualized rehabilitation programs for children with CP.

For other outcomes, MCID estimates remain limited, inconsistent, or insufficiently generalizable. Published MCID estimates for GMFM outcomes range from approximately 0.1% to 3.0% for the total score, 0.8% to 5.2% for Dimension D, and 0.3% to 4.9% for Dimension E; however, these estimates vary across analytic methods, GMFCS levels, and clinical settings. Therefore, although significant improvements were observed in Dimensions D and E, direct comparison with these thresholds requires consideration of the scoring system and population characteristics (114). For the MAS, QUEST, ABILHAND-Kids, and WeeFIM, available thresholds are either insufficiently established or not broadly applicable across pediatric CP populations (118–120). The improvement in ABILHAND-Kids detected only at follow-up of ≥6 weeks may suggest that some functional benefits become more apparent over time, highlighting the value of assessment beyond the immediate post-intervention period. Future studies should establish and validate outcome-specific MCIDs across different GMFCS levels and clinical contexts.

Several limitations of the present study warrant acknowledgement. The included trials exhibited heterogeneity in intervention parameters, and moderate to substantial statistical heterogeneity was observed for certain outcomes. Interventions varied in mechanisms of action and training protocols, and participants spanned a broad spectrum of age, CP subtype, and GMFCS level across trials. The limited number of included trials precluded additional stratified analyses by these patient-related factors. Thus, pooled effect sizes merely reflect the overall average benefit of emerging rehabilitation technologies rather than uniform efficacy across all intervention types and patient subgroups. Sensitivity analyses suggested that no single study unduly influenced the principal findings. The predominance of open-label designs without blinded outcome assessors introduces a risk of performance and detection bias, and the limited number of studies with extended follow-up constrains evaluation of long-term durability. Furthermore, several upper-extremity and secondary outcomes had limited statistical power. Finally, publication bias was assessed for outcomes with sufficient studies. Although Egger’s test suggested potential asymmetry for the 6MWT, the trim-and-fill analysis did not impute missing studies and the adjusted result remained unchanged, suggesting that the pooled estimate was not materially altered in this analysis.

Future high-quality RCTs should standardize intervention dosing, extend follow-up, and explore individualized strategies tailored to children across different GMFCS levels to strengthen the evidence base for emerging technologies in pediatric CP rehabilitation.

## 6 Conclusion

This meta-analysis demonstrates that emerging rehabilitation technologies confer significant short-term benefits for gait, gross motor function, balance, and functional independence in children with CP, with RAGT favoring walking speed and endurance, and VR favoring balance and standing function. Upper-limb spasticity and dissociated movement improved, while hand function gains exhibited delayed emergence at medium- to long-term follow-up. Most motor benefits were confined to the immediate post-intervention period, with only balance and hand function persisting beyond 6 weeks, underscoring the importance of sustained rehabilitation to consolidate long-term gains. The limited number of trials included for several outcomes weakens the external validity of pooled results, so our research conclusions should be generalized with caution. In light of between-study heterogeneity, limited long-term follow-up data, and measurement bias, these findings should be interpreted with caution. Future high-quality RCTs with extended follow-up are needed to verify long-term efficacy and strengthen individualized rehabilitation strategies for children with CP.

## Data Availability

The original contributions presented in the study are included in the article/Supplementary material, further inquiries can be directed to the corresponding authors.

## Supplementary material

SUPPLEMENTARY FIGURE S1 Forest plot of meta-analysis for QUEST dissociated movement score.

SUPPLEMENTARY FIGURE S2 Forest plot of meta-analysis for QUEST grasp score.

SUPPLEMENTARY FIGURE S3 Forest plot of meta-analysis for ABILHAND-Kids.

SUPPLEMENTARY FIGURE S4 Forest plot of the meta-analysis for WeeFIM total score.
