# Supplementary material for: Effectiveness of emerging technology-assisted rehabilitation in children with cerebral palsy: a systematic review and meta-analysis
Source: Front Neurol. 2026 Sep 9;17:1906886. doi: 10.3389/fneur.2026.1906886 (PMC13597381; doi:10.3389/fneur.2026.1906886)
Supplement: Supplementary file 1 [file Supplementary_file_1.DOCX]

**Appendix S1. Search strategies for electronic databases.**

**PubMed**

#1 Cerebral Palsy[MeSH Terms]

#2 "Spastic Cerebral Palsy"[Title/Abstract] OR "spastic diplegia*"[Title/Abstract] OR "spastic cerebral pals*"[Title/Abstract] OR "Rolandic Type Cerebral Palsy"[Title/Abstract] OR "Quadriplegic Infantile Cerebral Palsy"[Title/Abstract] OR "Monoplegic Cerebral Palsy"[Title/Abstract] OR "monoplegic cerebral pals*"[Title/Abstract] OR "Mixed Cerebral Palsy"[Title/Abstract] OR "Little's Disease"[Title/Abstract] OR "Little Disease"[Title/Abstract] OR "hypotonic cerebral pals*"[Title/Abstract] OR "Dyskinetic Cerebral Palsy"[Title/Abstract] OR "diplegia spastica"[Title/Abstract] OR "Congenital Cerebral Palsy"[Title/Abstract] OR "cerebral paresis"[Title/Abstract] OR "cerebral palsy*"[Title/Abstract] OR "central palsy*"[Title/Abstract] OR "Atonic Cerebral Palsy"[Title/Abstract] OR "Athetoid Cerebral Palsy"[Title/Abstract]

#3 "Robotics"[MeSH Terms]

#4 "Virtual Reality"[MeSH Terms])

#5 "Virtual Reality Exposure Therapy"[MeSH Terms]

#6 "Augmented Reality"[MeSH Terms]

#7 "Brain-Computer Interfaces"[MeSH Terms]

#8 "Digital Health"[MeSH Terms]

#9 "Micro-Electrical-Mechanical Systems"[MeSH Terms]

#10 "Intelligent Systems"[MeSH Terms]

#11 "Augmented Realities"[Title/Abstract] OR "Augmented Reality"[Title/Abstract] OR "BCI system"[Title/Abstract] OR "biofeedback software"[Title/Abstract] OR "BioMEMS"[Title/Abstract] OR "Brain Computer Interface"[Title/Abstract] OR "brain computer interface system"[Title/Abstract] OR "brain computer interface*"[Title/Abstract] OR "brain machine interface*"[Title/Abstract] OR "Companion Robot"[Title/Abstract] OR "companion robot*"[Title/Abstract] OR "Digital Health"[Title/Abstract] OR "Digital Health Technologies"[Title/Abstract] OR "Digital Health Technology"[Title/Abstract] OR "Digital Health"[Title/Abstract] OR "Digital Health Technologies"[Title/Abstract] OR "Digital Health Technology"[Title/Abstract] OR "direct neural interface"[Title/Abstract] OR "Educational Virtual Realities"[Title/Abstract] OR "Educational Virtual Reality"[Title/Abstract] OR "humanoid robot*"[Title/Abstract] OR "intelligent system*"[Title/Abstract] OR "MEMS"[Title/Abstract] OR "micro electrical mechanical system*"[Title/Abstract] OR "micro electro mechanical system*"[Title/Abstract] OR "mixed realit*"[Title/Abstract] OR "neural interface technolog*"[Title/Abstract] OR "RAGT"[Title/Abstract] OR "remote operation* robotic*"[Title/Abstract] OR "robot aided gait rehabilitation"[Title/Abstract] OR "robot aided gait training"[Title/Abstract] OR "robot assisted gait rehabilitation"[Title/Abstract] OR "robot assisted gait training"[Title/Abstract] OR "robot assisted procedure"[Title/Abstract] OR "robot assisted treatment"[Title/Abstract] OR "robotic aided gait training"[Title/Abstract] OR "robotic assisted gait rehabilitation"[Title/Abstract] OR "robotic assisted gait training"[Title/Abstract] OR "robotic assisted procedure"[Title/Abstract] OR "robotic assisted treatment"[Title/Abstract] OR "robotic gait training"[Title/Abstract] OR "robotically assisted procedure"[Title/Abstract] OR "Robotics"[Title/Abstract] OR "semi autonomous ai system*"[Title/Abstract] OR "smart device"[Title/Abstract] OR "smart equipment"[Title/Abstract] OR "smartdevice"[Title/Abstract] OR "social robot*"[Title/Abstract] OR "socially assistive robot*"[Title/Abstract] OR "soft robotic*"[Title/Abstract] OR "Telerobotics"[Title/Abstract] OR "Virtual Reality"[Title/Abstract] OR "Virtual Reality Exposure Therapy"[Title/Abstract] OR "Virtual Reality Immersion Therapy"[Title/Abstract] OR "virtual reality interface"[Title/Abstract] OR "virtual reality system"[Title/Abstract] OR "virtual reality therap*"[Title/Abstract] OR "Virtual Reality"[Title/Abstract] OR "Virtual Reality Immersion Therapy"[Title/Abstract] OR "virtual reality therap*"[Title/Abstract] OR "VR exposure therapy"[Title/Abstract] OR "VR interface"[Title/Abstract] OR "VR system"[Title/Abstract] OR "VRET"[Title/Abstract]

Search: (#1 OR #2) AND (#3 OR #4 OR #5 OR #6 OR #7 OR #8 OR #9 OR #10 OR #11) Filters: Randomized Controlled Trial Sort by: Most Recent

**Cochrane Library**

#1 (Spastic Cerebral Palsy):ti,ab,kw OR (Spastic Diplegia*):ti,ab,kw OR (Spastic Cerebral Pals*):ti,ab,kw OR (Rolandic Type Cerebral Palsy):ti,ab,kw OR (Quadriplegic Infantile Cerebral Palsy):ti,ab,kw OR (Monoplegic Cerebral Palsy):ti,ab,kw OR (Monoplegic Infantile Cerebral Palsy):ti,ab,kw OR (Monoplegic Cerebral Pals*):ti,ab,kw OR (Mixed Cerebral Palsy):ti,ab,kw OR (Mixed Cerebral Palsies):ti,ab,kw OR (Little's Disease):ti,ab,kw OR (Little Disease):ti,ab,kw OR (Hypotonic Cerebral Pals*):ti,ab,kw OR (encephalopathia infantilis):ti,ab,kw OR (Dystonic-Rigid Cerebral Pals*):ti,ab,kw OR (Dyskinetic Cerebral Palsy):ti,ab,kw OR (Diplegic Infantile Cerebral Palsy):ti,ab,kw OR (diplegia spastica):ti,ab,kw OR (Congenital Cerebral Palsy):ti,ab,kw OR (cerebral paresis):ti,ab,kw OR (cerebral palsy*):ti,ab,kw OR (central palsy*):ti,ab,kw OR (brain palsy*):ti,ab,kw OR (Atonic Cerebral Palsy):ti,ab,kw OR (Athetoid Cerebral Palsy):ti,ab,kw

#2 MeSH descriptor:[Cerebral Palsy] explode all trees

#3 MeSH descriptor:[Robotics] explode all trees

#4 MeSH descriptor:[Virtual Reality] explode all trees

#5 MeSH descriptor:[Virtual Reality Exposure Therapy] explode all trees

#6 MeSH descriptor:[Augmented Reality] explode all trees

#7 MeSH descriptor:[Brain-Computer Interfaces] explode all trees

#8 MeSH descriptor:[Micro-Electrical-Mechanical Systems] explode all trees

#9 MeSH descriptor:[Intelligent Systems] explode all trees

#10 (Augmented Realities):ti,ab,kw OR (Augmented Reality):ti,ab,kw OR (BCI controlled neuroprosthetic):ti,ab,kw OR (BCI system):ti,ab,kw OR (biofeedback software):ti,ab,kw OR (biofeedback system application software):ti,ab,kw OR (biofeedback system software):ti,ab,kw OR (BioMEMS):ti,ab,kw OR (Brain Computer Interface):ti,ab,kw OR (brain computer interface system):ti,ab,kw OR (Brain Computer Interface*):ti,ab,kw OR (brain computer interfacing system):ti,ab,kw OR (brain computing interface):ti,ab,kw OR (Brain Machine Interface*):ti,ab,kw OR (cerebellum machine interface):ti,ab,kw OR (cerebral computer interfaces):ti,ab,kw OR (Companion Robot):ti,ab,kw OR (Companion Robot*):ti,ab,kw OR (Digital Health):ti,ab,kw OR (Digital Health Technologies):ti,ab,kw OR (Digital Health Technology):ti,ab,kw OR (Digital Health):ti,ab,kw OR (Digital Health Technologies):ti,ab,kw OR (Digital Health Technology):ti,ab,kw OR (direct neural interface):ti,ab,kw OR (Educational Virtual Realities):ti,ab,kw OR (Educational Virtual Reality):ti,ab,kw OR (EEGer4):ti,ab,kw OR (Humanoid Robot*):ti,ab,kw OR (Instructional Virtual Realit*):ti,ab,kw OR (Instructional Virtual Reality):ti,ab,kw OR (Intelligent System*):ti,ab,kw OR (MedPresence):ti,ab,kw OR (MEMS):ti,ab,kw OR (Micro Electrical Mechanical System*):ti,ab,kw OR (Micro Electro Mechanical System*):ti,ab,kw OR (mind machine interface):ti,ab,kw OR (Mixed Realit*):ti,ab,kw OR (Neural Interface Technolog*):ti,ab,kw OR (RAGT):ti,ab,kw OR (Remote Operation* Robotic*):ti,ab,kw OR (robot aided gait rehabilitation):ti,ab,kw OR (robot aided gait therapy):ti,ab,kw OR (robot aided gait training):ti,ab,kw OR (robot aided procedure):ti,ab,kw OR (robot aided treatment):ti,ab,kw OR (robot assist gait training):ti,ab,kw OR (robot assisted gait rehabilitation):ti,ab,kw OR (robot assisted gait retraining):ti,ab,kw OR (robot assisted gait training):ti,ab,kw OR (robot assisted procedure):ti,ab,kw OR (robot assisted treatment):ti,ab,kw OR (robotic aided gait therapy):ti,ab,kw OR (robotic aided gait training):ti,ab,kw OR (robotic assisted gait rehabilitation):ti,ab,kw OR (robotic assisted gait training):ti,ab,kw OR (robotic assisted procedure):ti,ab,kw OR (robotic assisted treatment):ti,ab,kw OR (robotic gait training):ti,ab,kw OR (robotically assisted procedure):ti,ab,kw OR (robotically assisted treatment):ti,ab,kw OR (Robotics):ti,ab,kw OR (robotics aided gait training):ti,ab,kw OR (robotics assisted gait rehabilitation):ti,ab,kw OR (Semi Autonomous AI System*):ti,ab,kw OR (Semi Autonomous Artificial Intelligent Systems):ti,ab,kw OR (semi autonomous artificialsemi autonomous artificial intelligent systems):ti,ab,kw OR (smart device):ti,ab,kw OR (smart equipment):ti,ab,kw OR (smartdevice):ti,ab,kw OR (Social Robot*):ti,ab,kw OR (Socially Assistive Robot*):ti,ab,kw OR (Soft Robotic*):ti,ab,kw OR (Telerobotics):ti,ab,kw OR (Virtual Reality):ti,ab,kw OR (Virtual Reality Exposure Therapy):ti,ab,kw OR (Virtual Reality Immersion Therapy):ti,ab,kw OR (virtual reality interface):ti,ab,kw OR (virtual reality system):ti,ab,kw OR (Virtual Reality Therap*):ti,ab,kw OR (Virtual Reality):ti,ab,kw OR (Virtual Reality Immersion Therapy):ti,ab,kw OR (Virtual Reality Therap*):ti,ab,kw OR (VR exposure therapy):ti,ab,kw OR (VR immersion therapy):ti,ab,kw OR (VR interface):ti,ab,kw OR (VR system):ti,ab,kw OR (VRET):ti,ab,kw

#11 randomized controlled trail

#12 (#1 OR #2) AND (#2 OR #3 OR #4 OR #5 OR #6 OR #7 OR #8 OR #9 OR #10) AND #22

**Embase**

#1 'cerebral palsy'/exp OR 'cerebral palsy'

#2 'robotics'/exp OR 'robotics'

#3 'robot-assisted gait training'/exp OR 'robot-assisted gait training' OR 'robot-assisted procedure'/exp OR 'robot-assisted procedure'

#4 'virtual reality system'/exp OR 'virtual reality system'

#5 'virtual reality exposure therapy'/exp OR 'virtual reality exposure therapy'

#6 'augmented reality'/exp OR 'augmented reality'

#7 'brain computer interface'/exp OR 'brain computer interface'

#8 'digital health'/exp OR 'digital health'

#9 'biofeedback software (device)'/exp OR 'biofeedback software (device)'

#10 'smart device'/exp OR 'smart device'

#11 #2 OR #3 OR #4 OR #5 OR #6 OR #7 OR #8 OR #9 OR #10

#12 #1 AND #11

#13 'randomized controlled trial'/exp OR 'randomized controlled trial'

#14 #12 AND #13

**Web of Science (WOS)**

TS=(Robotics OR Virtual Reality OR Virtual Reality Exposure Therapy OR Brain-Computer Interface OR Digital Health OR Micro-Electrical-Mechanical Systems OR Intelligent Systems OR Augmented Realities OR Augmented Reality OR BCI controlled neuroprosthetic OR BCI system OR biofeedback software OR biofeedback system application software OR biofeedback system software OR BioMEMS OR Brain Computer Interface OR brain computer interface system OR Brain Computer Interface* OR brain computer interfacing system OR brain computing interface OR Brain Machine Interface* OR cerebellum machine interface OR cerebral computer interfaces OR Companion Robot OR Companion Robot* OR Digital Health OR Digital Health Technologies OR Digital Health Technology OR Digital Health OR Digital Health Technologies OR Digital Health Technology OR direct neural interface OR Educational Virtual Realities OR Educational Virtual Reality OR eegers OR Humanoid Robot* OR Instructional Virtual Realit* OR Instructional Virtual Reality OR Intelligent System* OR metapresence OR MEMS OR Micro Electrical Mechanical System* OR Micro Electro Mechanical System* OR mind machine interface OR Mixed Realit* OR Neural Interface Technolog* OR RAGT OR Remote Operation* Robotic* OR robot aided gait rehabilitation OR robot aided gait therapy OR robot aided gait training OR robot aided procedure OR robot aided treatment OR robot assist gait training OR robot assisted gait rehabilitation OR robot assisted gait retraining OR robot assisted gait training OR robot assisted procedure OR robot assisted treatment OR robotic aided gait therapy OR robotic aided gait training OR robotic assisted gait rehabilitation OR robotic assisted gait training OR robotic assisted procedure OR robotic assisted treatment OR robotic gait training OR robotically assisted procedure OR robotically assisted treatment OR Robotics OR robotics aided gait training OR robotics assisted gait rehabilitation OR Semi Autonomous AI System* OR Semi Autonomous Artificial Intelligent Systems OR semi autonomous artificialsei autonomous artificial intelligent systems OR smart device OR smart equipment OR smartdevices OR Social Robot* OR Socially Assistive Robot* OR Soft Robotic* OR Telerobotics OR Virtual Reality OR Virtual Reality Exposure Therapy OR Virtual Reality Immersion Therapy OR virtual reality interface OR virtual reality system OR Virtual Reality Therap* OR Virtual Reality OR Virtual Reality Immersion Therapy OR Virtual Reality Therap* OR VR exposure therapy OR VR immersion therapy OR VR interface OR VR system OR VRET) AND TS=(cerebral palsy OR Spastic Cerebral Palsy OR Spastic Diplegia* OR Spastic Cerebral Pals* OR Rolandic Type Cerebral Palsy OR Quadriplegic Infantile Cerebral Palsy OR monoplegia Cerebral Palsy OR monoplegia Infantile Cerebral Palsy OR monoplegia Cerebral Pals* OR Mixed Cerebral Palsy OR Mixed Cerebral Palsies OR littler Disease OR Little Disease OR Hypotonic Cerebral Pals* OR encephalopathic infantilis OR Dystonic-Rigid Cerebral Pals* OR Dyskinetic Cerebral Palsy OR Diplegic Infantile Cerebral Palsy OR diplegia spastics OR Congenital Cerebral Palsy OR cerebral paresis OR cerebral palsy* OR central palsy* OR brain palsy* OR Atonic Cerebral Palsy OR Athetoid Cerebral Palsy) AND TS=(randomized controlled trial)
