# Supplementary material for: Effectiveness of emerging technology-assisted rehabilitation in children with cerebral palsy: a systematic review and meta-analysis
Source: Front Neurol. 2026 Sep 9;17:1906886. doi: 10.3389/fneur.2026.1906886 (PMC13597381; doi:10.3389/fneur.2026.1906886)
Supplement: Supplementary file 2 [file Supplementary_file_2.DOCX]

**Appendix S2. Assessment of Publication Bias**

Publication bias was assessed visually using funnel plots and statistically using Egger’s regression test for outcomes with at least 10 studies. Analyses were conducted using Stata 18.0.

| **Outcome Measure** | **K** | **N** | **MD (95% CI)** | **p** | **Heterogeneity** | | **Publication Bias** | **Trim and Fill** | |
| --- | --- | --- | --- | --- | --- | --- | --- | --- | --- |
|  |  |  |  |  | **Q (df)** | **I² (%) (p)** | **Egger p** | **Adj Effect Size** | **% var** |
| **Lower Limb Function** | | | | | | | | | |
| 6MWT (m) | 10 | 414 | 43.56 [25.55, 61.57] | <0.00001 | 16.12 (9) | 44% (0.06) | 0.014 | 43.56 [25.55, 61.57] | 0% |
| GMFM-dimension D (%) | 10 | 345 | 5.24 [1.53, 8.95] | 0.006 | 27.71 (9) | 68% (0.001*) | 0.199 | - | - |
| GMFM-dimension E (%) | 10 | 345 | 6.66 [1.95, 11.38] | 0.006 | 33.36 (9) | 73% (0.0001*) | 0.184 | - | - |
| PBS (points) | 10 | 344 | 5.21 [4.06, 6.37] | <0.00001 | 7.23 (9) | 0% (0.61) | 0.480 | - | - |

**Figure S2.1. Egger's regression test for publication bias in the 6-Minute Walk Test (6MWT) (P = 0.014).**

**Figure S2. Trim-and-fill funnel plot for the 6-Minute Walk Test. The analysis imputed zero missing studies, confirming the robustness of the result.**

**Figure S2.2. Egger's test for publication bias in GMFM Dimension D (P = 0.199).**

**Figure S2.3. Egger's test for publication bias in GMFM Dimension E (P = 0.184).**

**Figure S2.4. Egger's test for publication bias in PBS (P = 0.480).**
