# Supplementary material for: Effectiveness of emerging technology-assisted rehabilitation in children with cerebral palsy: a systematic review and meta-analysis
Source: Front Neurol. 2026 Sep 9;17:1906886. doi: 10.3389/fneur.2026.1906886 (PMC13597381; doi:10.3389/fneur.2026.1906886)
Supplement: Supplementary file 3 [file Supplementary_file_3.docx]

**Table S3.1 GRADE Summary of Findings: lower-limb gait, gross motor and balance outcomes**

| **Certainty assessment** | | | | | | | **№ of patients** | | **Effect** | | **Certainty** | **Importance** |
| --- | --- | --- | --- | --- | --- | --- | --- | --- | --- | --- | --- | --- |
| **№ of studies** | **Study design** | **Risk of bias** | **Inconsistency** | **Indirectness** | **Imprecision** | **Other considerations** | **Emerging technology** | **Conventional rehabilitation** | **Relative (95% CI)** | **Absolute (95% CI)** |  |  |
| **10MWT (assessed with: 10-meter walk test; time taken to complete 10 meters, unit: seconds (s))** | | | | | | | | | | | | |
| 6 | randomised trials | not serious | not serious | not serious | serious^a^ | none | 86 | 86 | - | MD **2.83 s lower** (4.39 lower to 1.27 lower) | ⨁⨁⨁◯ Moderate^a^ | CRITICAL |
| **6MWT (assessed with: 6-minute walk test; total walking distance within 6 minutes, unit: meters (m))** | | | | | | | | | | | | |
| 10 | randomised trials | serious^b^ | not serious | not serious | not serious | none | 208 | 206 | - | MD **43.56 m higher** (22.55 higher to 61.57 higher) | ⨁⨁⨁◯ Moderate^b^ | CRITICAL |
| **GMFM-Total (assessed with: GMFM Total for gross motor function, unit: %)** | | | | | | | | | | | | |
| 8 | randomised trials | serious^b^ | not serious | not serious | serious^a^ | none | 145 | 146 | - | MD **2.22 % higher** (0.62 lower to 5.07 higher) | ⨁⨁◯◯ Low^a,b^ | CRITICAL |
| **GMFM-dimension D (assessed with: GMFM-dimension D (standing function), unit: %)** | | | | | | | | | | | | |
| 10 | randomised trials | not serious | serious^c^ | not serious | serious^a^ | none | 172 | 173 | - | MD **5.24 % higher** (1.53 higher to 8.95 higher) | ⨁⨁◯◯ Low^a,c^ | CRITICAL |
| **GMFM-dimension E (assessed with: GMFM-dimension E (walking, running and jumping), unit: %)** | | | | | | | | | | | | |
| 10 | randomised trials | not serious | serious^d^ | not serious | serious^a^ | none | 172 | 173 | - | MD **6.66 % higher** (1.95 higher to 11.38 higher) | ⨁⨁◯◯ Low^a,d^ | CRITICAL |
| **TUG (assessed with: Timed Up and Go functional mobility test, unit: seconds(s))** | | | | | | | | | | | | |
| 3 | randomised trials | not serious | serious^e^ | not serious | serious^a^ | none | 39 | 39 | - | MD **2.17 s lower** (3.6 lower to 0.75 lower) | ⨁⨁◯◯ Low^a,e^ | CRITICAL |
| **PBS (assessed with: Pediatric Balance Scale balance function score, unit: points)** | | | | | | | | | | | | |
| 10 | randomised trials | not serious | not serious | not serious | serious^a^ | none | 169 | 175 | - | MD **5.21 points higher** (4.06 higher to 6.37 higher) | ⨁⨁⨁◯ Moderate^a^ | CRITICAL |

**CI:** confidence interval; **MD:** mean difference

#### Explanations

a. Certainty was downgraded due to serious imprecision: the total pooled sample size fell below the GRADE-recommended optimal information size (OIS) threshold of 400 for continuous outcomes, leading to relatively wide 95% confidence intervals.
b. Among the included RCTs, several trials adopted open-label designs without blinded outcome assessors, creating potential measurement bias.
c. Primary analysis showed high heterogeneity (I²=68%), mainly driven by one outlier trial (Fu2022, I² fell to 12% after removal). Differences in baseline motor severity, intervention schedules, and participant characteristics may also have contributed to the observed inconsistency.
d. Primary meta-analysis showed high inter-study heterogeneity (I²=73%). Sensitivity analysis confirmed that the major source of inconsistency was the trial Yenilmez 2025; heterogeneity dropped to I²=33% after excluding this outlying study. Subgroup analyses stratified by intervention duration and types of emerging rehabilitation technology were conducted to explore variance sources, but moderate residual heterogeneity still existed within subgroups. Additional contributors included large disparities in baseline walking ability across participants.

e. Moderate inter-study heterogeneity (I²=50%) was detected. Subgroup analyses stratified by intervention duration and types of emerging rehabilitation technology could not fully eliminate residual heterogeneity. Key contributors to inconsistency included the divergent initial mobility impairment severity of children with cerebral palsy, as well as different GMFM levels at enrolment across trials.

**Table S3.2 GRADE Summary of Findings: lower-limb spasticity and functional independence outcomes**

| **Certainty assessment** | | | | | | | **№ of patients** | | **Effect** | | **Certainty** | **Importance** |
| --- | --- | --- | --- | --- | --- | --- | --- | --- | --- | --- | --- | --- |
| **№ of studies** | **Study design** | **Risk of bias** | **Inconsistency** | **Indirectness** | **Imprecision** | **Other considerations** | **Emerging technology** | **Conventional rehabilitation** | **Relative (95% CI)** | **Absolute (95% CI)** |  |  |
| **MAS-Lower limb (assessed with: Modified Ashworth Scale for lower limb spasticity, unit: grade)** | | | | | | | | | | | | |
| 4 | randomised trials | not serious | very serious^a^ | not serious | serious^b^ | none | 62 | 59 | - | MD **0.53 grade lower** (1.22 lower to 0.17 higher) | ⨁◯◯◯ Very low^a,b^ | CRITICAL |
| **WeeFIM Total-Lower limb (assessed with: Wee Functional Independence Measure total score, unit: points)** | | | | | | | | | | | | |
| 6 | randomised trials | not serious | not serious | not serious | serious^b^ | none | 105 | 105 | - | MD **6.71 points higher** (2.94 higher to 10.49 higher) | ⨁⨁⨁◯ Moderate^b^ | IMPORTANT |
| **WeeFIM Self-care-Lower limb (assessed with: Wee Functional Independence Measure self-care ability score, unit: points)** | | | | | | | | | | | | |
| 3 | randomised trials | not serious | not serious | not serious | serious^b^ | none | 65 | 65 | - | MD **3.22 points higher** (0.72 higher to 5.73 higher) | ⨁⨁⨁◯ Moderate^b^ | IMPORTANT |

**CI:** confidence interval; **MD:** mean difference

#### Explanations

a. Extremely high inter-study heterogeneity (I²=87%) was detected across trials. Subgroup analyses stratified by intervention duration and rehabilitation technology types could not eliminate severe residual variance. Major contributors to inconsistency included large differences in initial lower limb spasticity severity and divergent baseline MAS scores at enrollment.

b. Certainty was downgraded due to serious imprecision: the total pooled sample size fell below the GRADE-recommended optimal information size (OIS) threshold of 400 for continuous outcomes, leading to relatively wide 95% confidence intervals.

**Table S3.3 GRADE Summary of Findings: Upper-extremity manual function outcomes**

| **Certainty assessment** | | | | | | | **№ of patients** | | **Effect** | | **Certainty** | **Importance** |
| --- | --- | --- | --- | --- | --- | --- | --- | --- | --- | --- | --- | --- |
| **№ of studies** | **Study design** | **Risk of bias** | **Inconsistency** | **Indirectness** | **Imprecision** | **Other considerations** | **Emerging technology** | **Conventional rehabilitation** | **Relative (95% CI)** | **Absolute (95% CI)** |  |  |
| **MAS-Upper limb (assessed with: Modified Ashworth Scale, unit: grade)** | | | | | | | | | | | | |
| 2 | randomised trials | not serious | not serious | not serious | serious^a^ | none | 35 | 35 | - | MD **0.4 grade lower** (0.59 lower to 0.21 lower) | ⨁⨁⨁◯ Moderate^a^ | CRITICAL |
| **QUEST-Total (assessed with: Quality of Upper Extremity Skills Test, unit: %)** | | | | | | | | | | | | |
| 2 | randomised trials | not serious | very serious^b^ | not serious | serious^a^ | none | 24 | 24 | - | MD **1.8 %**  **lower** (18.77 lower to 15.17 higher) | ⨁◯◯◯ Very low^a,b^ | IMPORTANT |
| **QUEST-Dissociated movement (assessed with: Quality of Upper Extremity Skills Test-Dissociated movement,unit: %)** | | | | | | | | | | | | |
| 3 | randomised trials | not serious | serious^c^ | not serious | serious^a^ | none | 53 | 53 | - | MD **4.67 % higher** (0.99 higher to 8.35 higher) | ⨁⨁◯◯ Low^a,c^ | IMPORTANT |
| **QUEST-Grasp (assessed with: Quality of Upper Extremity Skills Test-Grasp,unit: %)** | | | | | | | | | | | | |
| 4 | randomised trials | not serious | serious^d^ | not serious | serious^a^ | none | 62 | 62 | - | MD **2.59 % higher** (3.41 lower to 8.58 higher) | ⨁⨁◯◯ Low^a,d^ | IMPORTANT |
| **ABILHAND-Kids (assessed with: ABILHAND-Kids questionnaire for children's upper limb manual ability, unit: points)** | | | | | | | | | | | | |
| 5 | randomised trials | not serious | not serious | not serious | serious^a^ | none | 61 | 60 | - | MD **2 points higher** (1.01 lower to 5.01 higher) | ⨁⨁⨁◯ Moderate^a^ | CRITICAL |
| **BBT (assessed with: Box and Blocks Test , unit: blocks/min)** | | | | | | | | | | | | |
| 4 | randomised trials | not serious | not serious | not serious | serious^a^ | none | 47 | 46 | - | MD **1.13 blocks/min higher** (3.27 lower to 5.53 higher) | ⨁⨁⨁◯ Moderate^a^ | IMPORTANT |
| **AHA (assessed with: Assisting Hand Assessment, unit: points)** | | | | | | | | | | | | |
| 2 | randomised trials | not serious | not serious | not serious | serious^a^ | none | 33 | 37 | - | MD **4.24 points lower** (12.21 lower to 3.74 higher) | ⨁⨁⨁◯ Moderate^a^ | CRITICAL |

**CI:** confidence interval; **MD:** mean difference

#### Explanations

a. Certainty was downgraded due to serious imprecision: the total pooled sample size fell below the GRADE-recommended optimal information size (OIS) threshold of 400 for continuous outcomes, leading to relatively wide 95% confidence intervals.

b. Extremely high inter-study heterogeneity (I²=83%) was detected across the two trials. Subgroup analyses stratified by intervention duration and rehabilitation technology types failed to eliminate severe residual variance. Key contributors to inconsistency included wide disparities in participants’ baseline age, large differences in initial upper extremity function impairment severity, and significant divergence in intervention protocols between the two studies.

c. Initial pooled analysis showed extremely high inter-study heterogeneity (I²=87%). Sensitivity analysis confirmed that the trial El-Shamy (2018) was the dominant source of heterogeneity; excluding this outlying study reduced I² to 0%, and the overall pooled conclusion remained unchanged. Subgroup analyses stratified by intervention duration and rehabilitation technology types were performed to explore variance sources. Minor between-study discrepancies were attributed to wide variation in participants’ baseline age and divergent initial upper limb isolated motor impairment severity at enrolment.

d. The primary pooled meta-analysis showed extremely high inter-study heterogeneity (I²=91%). Sensitivity analysis confirmed El-Shamy (2018) was the sole outlier driving severe inconsistency; excluding this study reduced I² to 0%, and the overall pooled statistical conclusion remained unchanged. Residual between-study discrepancies were mainly attributed to wide variation in participants’ baseline age and markedly divergent initial grasping function impairment severity at enrolment.

**Table S3.4 GRADE Summary of Findings: Global function and occupational participation outcomes**

| **Certainty assessment** | | | | | | | **№ of patients** | | **Effect** | | **Certainty** | **Importance** |
| --- | --- | --- | --- | --- | --- | --- | --- | --- | --- | --- | --- | --- |
| **№ of studies** | **Study design** | **Risk of bias** | **Inconsistency** | **Indirectness** | **Imprecision** | **Other considerations** | **Emerging technology** | **Conventional rehabilitation** | **Relative (95% CI)** | **Absolute (95% CI)** |  |  |
| **WeeFIM Total (assessed with: Wee Functional Independence Measure total score for overall functional independence, unit: points)** | | | | | | | | | | | | |
| 7 | randomised trials | not serious | not serious | not serious | serious^a^ | none | 114 | 115 | - | MD **6.67 points higher** (2.97 higher to 10.37 higher) | ⨁⨁⨁◯ Moderate^a^ | IMPORTANT |
| **COPM Performance (assessed with: Canadian Occupational Performance Measure performance score, unit: points)** | | | | | | | | | | | | |
| 3 | randomised trials | not serious | not serious | not serious | serious^a^ | none | 53 | 57 | - | MD **0.14 points higher** (0.37 lower to 0.65 higher) | ⨁⨁⨁◯ Moderate^a^ | IMPORTANT |

**CI:** confidence interval; **MD:** mean difference

#### Explanations

a. Certainty was downgraded due to serious imprecision: the total pooled sample size fell below the GRADE-recommended optimal information size (OIS) threshold of 400 for continuous outcomes, leading to relatively wide 95% confidence intervals.
